# A Methodological Approach to Small Area Estimation for the Behavioral Risk Factor Surveillance System

**DOI:** 10.5888/pcd13.150480

**Published:** 2016-07-14

**Authors:** Carol Pierannunzi, Fang Xu, Robyn C. Wallace, William Garvin, Kurt J. Greenlund, William Bartoli, Derek Ford, Paul Eke, G. Machell Town

**Affiliations:** Author Affiliations: Fang Xu, William Garvin, G. Machell Town, Division of Population Health, Population Health Surveillance Branch, Centers for Disease Control and Prevention, Atlanta, Georgia; Robyn C. Wallace, William Bartoli, Northrup Grumman, Atlanta, Georgia; Kurt J. Greenlund, Paul Eke, Office of the Director, Centers for Disease Control and Prevention, Atlanta, Georgia; Derek Ford, National Center for Injury Prevention and Control, Division of Violence Prevention, Centers for Disease Control and Prevention, Atlanta, Georgia.

## Abstract

Public health researchers have used a class of statistical methods to calculate prevalence estimates for small geographic areas with few direct observations. Many researchers have used Behavioral Risk Factor Surveillance System (BRFSS) data as a basis for their models. The aims of this study were to 1) describe a new BRFSS small area estimation (SAE) method and 2) investigate the internal and external validity of the BRFSS SAEs it produced. The BRFSS SAE method uses 4 data sets (the BRFSS, the American Community Survey Public Use Microdata Sample, Nielsen Claritas population totals, and the Missouri Census Geographic Equivalency File) to build a single weighted data set. Our findings indicate that internal and external validity tests were successful across many estimates. The BRFSS SAE method is one of several methods that can be used to produce reliable prevalence estimates in small geographic areas.

## Introduction

Whereas public health data are commonly collected nationally, public health policy is implemented primarily at the local level. In many instances, state and local governments use geographically based information in health policy planning and program implementation (1,2). Research indicates that social, cultural, and community characteristics are determinants of health behaviors specific to local jurisdictions (1). Although national and state data can inform local planning, there is an increasing need for information specific to smaller geographic areas. In response, public health agencies and researchers have used small area estimation (SAE) methods that use state or national data to model reliable prevalence estimates.

The Behavioral Risk Factor Surveillance System (BRFSS) has collected state-level public health information since 1984 using a system of telephone surveys in all 50 states, Washington, DC, and US territories (3), conducting more than 400,000 interviews annually. The BRFSS provided limited SAEs through Selected Metropolitan/Micropolitan Area Risk Trends (SMART), which produced direct estimates where sample sizes were more than 500 (4). BRFSS data have been used to model health status and health risk behaviors for substate geographic areas within single states. Such investigations included a study of asthma in Massachusetts (5) and obesity in Mississippi (6). Researchers also have used BRFSS data to model SAEs for a single chronic condition across all US counties (7–9). Many large-scale efforts combine data from several databases, including the BRFSS, to provide SAEs for public health planning and action across all counties (10–12).

Given the advancements in SAE, it is not surprising that researchers have applied many methods to produce estimates. To respond to the demand for SAEs from the BRFSS, we developed a method that is aligned with BRFSS methods and inclusive of all counties. Many criteria were established for the BRFSS SAE method. It was necessary to incorporate cellular telephone samples and a new weighting method called *iterative proportional fitting* or “raking,” which have been part of the BRFSS since 2011 (13,14). Additionally, the method had to rely on state-level rather than national-level weighting. Finally, the method had to be one that could be internally and externally validated. Internal validity was defined as the ability of the method to reproduce point estimates created by direct BRFSS prevalence from the same period. External validity was defined as the ability of the method to produce similar estimates to those of other sources. The aims of this study were to 1) describe the BRFSS SAE method and 2) investigate the internal and external validity of estimates produced.

## Methods

The BRFSS SAE method relied on data from 4 sources: 1) the 2013 BRFSS (n = approximately 430,000), which provides data on health behaviors, demographic characteristics, and county (3); 2) the 2008–2012 American Community Survey (ACS) Public Use Microdata Sample (PUMS) (15), which provided individual-level data with person weights (n = approximately 11,600,000) and Public Use Microdata Areas (PUMAs); 3) the Missouri Census Geographic Equivalency File (GEOCORR), which matched counties and PUMAs (15); and 4) the 2013 Nielsen Claritas population totals used for weighting to county-level populations (16).

The GEOCORR provided the connection between county and PUMAs and permitted health information in the BRFSS to be connected to locational information in the ACS PUMS. PUMAs are based on population size, are generally constructed on jurisdictional boundaries, and do not cross state lines. Typically, counties with large populations are subdivided into multiple PUMAs, while PUMAs in rural areas are made up of groups of counties. PUMAs can cross county boundaries, and a PUMA can be made up of parts of several different counties. Using the GEOCORR, a classification of counties-to-PUMAs was created that divided the counties into the following categories: 1) counties covered by 1 PUMA that did not cover any other counties (approximately 7% of all counties), 2) counties covered by 2 or more PUMAs that cover at least 1 other county (approximately 2.2% of all counties), 3) counties covered by 2 or more PUMAs that did not cover other counties (approximately 3.5% of all counties), and 4) all other counties (approximately 87% of all counties).

This classification did not determine an exact PUMA-to-county match except for categories 1 and 3, but it provided a correlation between PUMAs and counties, which was used to interpret locations of people within ACS PUMS data sets. In approximately 2.2% of cases (among those that fell into category 2), a weighting adjustment was needed to account for the known location of people and population estimates. This weight adjustment was necessary to make the total population of the PUMA match the known population of the county.

The method to produce the BRFSS SAE followed these steps:

1. **BRFSS 2013 data raking. **BRFSS data were weighted (3) using the same variables (age, race, sex, race/ethnicity, home ownership, marital status, education level, and telephone ownership) as the BRFSS public-use data sets.

2. **BRFSS imputation. **Missing outcomes (due to item nonresponse) in the BRFSS were imputed using the hot deck procedure in SAS (SAS Institute, Inc) and SUDAAN (RTI International) (17).

3. **1-Year ACS PUMS data created. **The single-year ACS PUMS data set was created from the multiple years of ACS data available. This data set provides the 2013 household and person population data needed to determine estimates for each county.

4. **The ACS PUMS county assignments made using the GEOCORR.** The GEOCORR PUMA assignments were matched to counties in the 4 PUMA categories. For 3 of those categories an exact or correlated location was assigned to each person in the ACS file. In approximately 2% of cases, the person weight was multiplied by the sample size of the county and PUMA combination then divided by the sum of the person weight in that PUMA.

5. **ACS PUMS data raked. **The ACS PUMS data were raked at county level using Nielsen Claritas control totals by age, sex, and race/ethnicity. Data were then raked at the state level using the same margins as BRFSS raking (3) except for telephone ownership (not available in ACS PUMS).

6. **ACS PUMS and BRFSS data sets stacked to prepare for modeling. **The 2 data sets were set together, using county as a key variable.

7. **Prevalence estimates generated.** A linear random effects model (LREM) for each indicator was run on the BRFSS data set, adjusting for age, race/ethnicity, and sex using county as a random effect. (Many models using different sets of variables were attempted. The equation with age, race/ethnicity, and sex was the most efficient and predictive of the tested models. Other variables tested in the models included education level, marital status, [Hispanic] ethnicity, and home ownership.) The model coefficients were applied to the ACS PUMS data set to generate the adjusted prevalence at the county level using PROC GLIMMIX in SAS (18). Predicted probabilities were computed using the random effect, the estimated best linear unbiased predictors, in the final linearized model. These predicted probabilities were then averaged by county to produce prevalence estimates for each of the counties in the data set.

The model specification was:


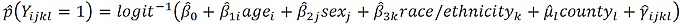


where i, j, k was the number of categories in each demographic variables, l denoted the county and 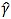 was the random residual normally distributed. Individual-level probabilities were aggregated to the county level to produce a county estimate.

## Validity Assessments

We conducted several internal validity checks of the SAEs. First, we compared the SAEs against BRFSS direct survey estimates for counties where there were sufficient responses (n = 500), traditionally provided in the BRFSS SMART. In 2013, 223 counties were identified with at least 500 responses ranging in population from approximately 16,000 to more than 100,000 residents in 47 states. Five general health and access-to-care indicators were used to test internal validity against the BRFSS SAE. These included the proportion of the population reporting fair or poor general health, the proportion reporting greater than 14 physically unhealthy days within the past month, the proportion reporting greater than 14 mentally unhealthy days within the last month, the proportion reporting delayed medical care due to cost within the past 12 months, and the proportion of the population that is uninsured (age 18–64). These were selected because of their low item nonresponse and their location in the core portion of the BRFSS survey. We compared individual county-level estimates and confidence intervals for health status and access indicators. However, confidence intervals for the 2 methods are derived in 2 different ways, making comparisons difficult. The BRFSS direct estimates include complex sample design probabilities in the calculations of confidence intervals. BRFSS SAE methods use the SAS GLIMMIX procedure, which does not allow for the sampling survey design variables (ie, strata variable and primary sampling unit). The weight statement in GLIMMIX is not a sampling weight but a frequency weight, which may underestimate the variance.

As a second internal validity check, we compared SAEs with each county in Florida (n = 67). In 2013, Florida adopted a county-based sample that allowed this comparison. We examined the same 5 indicators for the BRFSS direct estimates in the Florida counties and the BRFSS SAE for those counties. We then aggregated the counties to the state level and compared the aggregated direct estimates to aggregated SAEs for the state.

The proportion of the population (aged 18–64) without health insurance was used as a check of external validity. This was the only variable that was common to the BRFSS and the ACS. Both the BRFSS and the ACS (19) collected information on whether people were insured. However, the BRFSS used a single question and the ACS used a series of questions to ascertain whether respondents had insurance. The population base was also slightly different, because unlike the BRFSS, the ACS excluded active military personnel. The ACS also collected information from paper surveys, online questionnaires, by telephone, or in person and permitted proxy interviews, while the BRFSS was conducted exclusively by telephone without proxy interviews. Therefore, some differences in prevalence estimates were expected.

Because the ACS provided 1-, 3-, and 5-year estimates based on county population size, external validity checks of insurance coverage could be made only for 817 counties (with populations >65,000) where there was a 1-year estimate in 2013. Correlation coefficients and mean absolute differences between the estimates were also calculated.

## Results

Summary comparisons of prevalence estimates from the BRFSS direct estimates and the BRFSS SAEs for 5 variables among 223 counties were calculated (Table 1). In all 5 cases, means and medians were close. Differences, even at the minimum and maximum values, were approximately within one percentage point. Correlation coefficients indicated that the 2 measures had strong linearity and agreement (correlation coefficients ranged from .97 to .99; concordant coefficients ranged from .96 to .98). County-by-county estimates and confidence intervals for the same indicators are provided in the Appendix. For these counties, the BRFSS SAE indicator is consistently with the confidence interval of the direct estimate, although caution should be used in interpreting the 2 sets of confidence intervals. As noted, the SAS GLIMMIX procedure does not consider the complex sample of the BRFSS, which reduces the magnitude of the confidence intervals.

**Table 1:** Internal Validity Testing Using General Health Indicators, BRFSS Direct Estimates and BRFSS SAE (n = 223)

Summary Statistics	Estimate, %		Correlation Coefficient		
	BRFSS Direct	BRFSS SAE	Pearson	Spearman	Concordant
**Fair/poor health**					
Minimum	7.30	8.39	.98	.97	.97
25th percentile	12.96	13.37			
Median	16.10	16.04			
Mean	16.09	16.29			
75th percentile	18.90	18.85			
Maximum	31.28	31.06			
**>14 Physically unhealthy days in the past month**					
Minimum	4.77	5.23	.98	.98	.96
25th percentile	8.35	8.90			
Median	10.19	10.75			
Mean	10.32	10.68			
75th percentile	12.00	12.28			
Maximum	18.64	18.16			
**>14 Mentally unhealthy days in the past month**					
Minimum	5.08	5.34	.99	.98	.97
25th percentile	8.72	8.75			
Median	10.18	10.13			
Mean	10.24	10.14			
75th percentile	11.39	11.17			
Maximum	18.42	17.22			
**Delayed medical care due to cost in the past 12 months**					
Minimum	4.25	4.76	.99	.98	.98
25th percentile	11.93	11.43			
Median	15.29	14.19			
Mean	14.98	14.24			
75th percentile	17.88	16.82			
Maximum	34.33	33.01			
**Uninsured**					
Minimum	5.11	5.41	.99	.98	.97
25th percentile	14.19	13.59			
Median	19.46	18.26			
Mean	19.70	18.35			
75th percentile	24.25	22.50			
Maximum	57.52	56.11			

Abbreviations: BRFSS, Behavioral Risk Factor Surveillance System; SAE, small-area estimation.

When the same indicators were compared with direct estimates of all 67 counties in Florida (Table 2), they compared well for modeled and direct estimates for mean and median values. Differences were noted on more extreme values for 2 variables. No health insurance and delay of medical care each differed by 3 percentage points at the 75th percentile but were close in value to the direct estimate at the 25th percentile. When aggregated to the state level, the SAEs were different from the direct state estimate by about 1 percentage point for no health insurance to essentially no difference for greater than 14 mentally unhealthy days. A difference of more than 10 percentage points was noted at the maximum value for delayed medical care (at 29.99 and 41.96 for the BRFSS SAE and BRFSS direct, respectively). This outlier may have resulted from the low number of responses in that county. All other minimum and maximum values were within 2 percentage points.

**Table 2:** State-Level Estimates of BRFSS SAE and Direct Estimates From the 2013 Florida BRFSS

Estimate	No Health Insurance	Fair/Poor Health	>14 Physically Unhealthy Days in the Past Month	>14 Mentally Unhealthy Days in the Past Month	Delayed Medical Care Due to Cost in the Past 12 Months
	%				
**BRFSS SAE**					
Minimum	16.14	12.81	9.37	7.43	11.86
25th percentile	22.66	19.31	13.07	10.67	15.70
Median	26.24	21.44	14.41	12.19	18.45
Mean	27.03	22.13	14.97	12.72	18.73
75th percentile	30.52	24.96	16.63	14.08	20.60
Maximum	42.93	33.52	30.78	27.05	29.99
SAE state estimate, aggregated	27.53	19.96	13.77	11.91	19.70
**BRFSS direct**					
Minimum	14.73	10.96	7.63	6.67	10.37
25th percentile	22.85	18.95	12.11	10.42	16.31
Median	28.20	20.90	14.14	12.58	19.90
Mean	28.99	21.92	14.42	12.81	19.97
75th percentile	33.97	25.21	16.63	14.04	23.09
Maximum	45.26	34.15	29.46	24.94	41.96
BRFSS state estimate, direct	28.68	19.37	13.14	11.90	20.40

Abbreviations: BRFSS, Behavioral Risk Factor Surveillance System; SAE, small-area estimate.

For our external validity check, there were 817 counties with 1-year ACS estimates and BRFSS SAEs (Table 3). The modeled estimates were close to the ACS estimates in terms of the median and mean but differed on the maximum values. The minimum value differed by almost 2 percentage points (at 4.91 for the BRFSS SAE and 3.00 for the ACS). The correlation coefficient was .77 (*P* < .01). The mean absolute and relative differences between the BRFSS SAE and the 1-year ACS estimates were 3.54 percentage points and 20.22 percentage points, respectively. A scatterplot of data from individual counties is also provided (Figure). 

**Table 3:** Comparison of BRFSS SAE and ACS 1-Year Estimates of Uninsured Population by County 2013 (n = 817)

Summary Statistics	BRFSS SAE^a^	ACS Direct Estimate
Minimum	4.91	3.00
25th Percentile	13.64	13.50
Median	18.15	18.40
Mean	18.86	18.90
75th Percentile	23.08	23.50
Maximum	71.85	53.50
Pearson *r* (*P* value)	.77 (<.01)	
**Mean and absolute differences in estimates, percentage points**		
Mean absolute differences	3.54	
Mean relative differences	20.22	

Abbreviations: ACS, American Community Survey; BRFSS, Behavioral Risk Factor Surveillance System; SAE, small-area estimate.

a BRFSS SAEs were rounded to 2 decimals. A zero was placed in the hundredth column to of the ACS estimate to allow for comparison but should not be interpreted as part of the ACS estimate.

**Figure:**
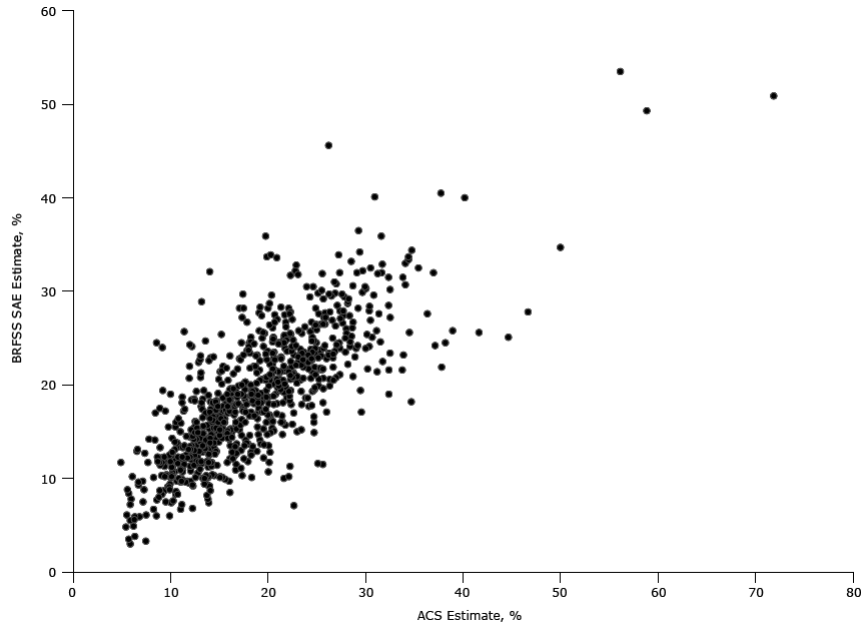
Behavioral Risk Factor Surveillance System (BRFSS) Small Area Estimation (SAE) and American Community Survey (ACS) Estimates of Uninsured Population Prevalence, 2013.

## Discussion and Conclusions

The methods described here were developed to provide estimates of health status and access indicators from the BRFSS that can be used by state and local health departments in a manner that is standardized within and across states and that is consistent with BRFSS methods at the state level. However, the method has some disadvantages. It is limited to items which are, or can be recoded into, dichotomous (yes/no) responses. The method is dependent on the link between the PUMA and the county, which is not a one-to-one relationship and which requires adjustment in the weighting in approximately 5% of cases. Moreover, the design weights are not incorporated into PROC GLIMMIX, making confidence intervals and standard errors unreliable. Furthermore, because the model relies on the BRFSS data, if any county in the BRFSS data set does not contain a single observation, the model cannot be used to compute an SAE for that county. The method may be adapted only to geographic areas that are composed of counties in a single state. For example, prevalence estimates for micropolitan/metropolitan statistical areas (MSAs) in a state could be calculated, but because state-level weights are used, the method could not be used if the MSA crossed state lines. Public health districts or other substate jurisdictions based on counties could also use the BRFSS SAE method. The LREM model used in this analysis may not include variables that could be significant predictors for health outcomes in specific counties, states, or both. Estimates may vary on the basis of mode of data collection, temporal differences, and other factors. In particular, we do not know the effect of local policies, practices, or interventions that could influence outcomes using modeled estimates. However, given resource constraints to collect quality, valid, and reliable data at the local level, methods using rigorously developed modeling can produce acceptable estimates for program planning. The BRFSS SAEs are modeled and may be different from direct survey estimates. Whether such modeling can be used for evaluation of programs or program outcomes has yet to be determined.

The BRFSS SAE method was built on years of research and findings within the Centers for Disease Control and Prevention and the extended literature. This system capitalized on contemporary population information made available in the ACS PUMS data set with the work of the Missouri Census Data Center (GEOCORR). Because GEOCORR allowed for a crosswalk of the PUMA to the county, the data set enhanced the accuracy of assigning individuals to counties. The advantages to this method were that weighting was more current to the population year and more specific to the location of the resident. The use of state-level weighting was a criterion of the methods under review. Rather than using national weighting, as is done in other SAE approaches, the state and the county populations became the targets for the weighting margins, aligning with the state-level focus of the BRFSS. The use of the ACS PUMS also meant that the weights could be adjusted annually. The LREM used in the estimation step of the BRFSS SAE system allowed for the inclusion of age, race/ethnicity, and sex as factors in the GLIMMIX model with county as a random effect. Other factors could be added to the model if needed. Age adjustment may be appropriate for cross-state or nationwide comparisons. Age standardization of the BRFSS SAE can be accomplished by using national population totals for a single year and adjusting the proportion of each age stratum.

This method fits into the landscape of approaches to SAE, each with unique strengths and purposes. We set out to meet the demands of our data users with specific criteria: we wanted to be sure that our method incorporated methods already in place (13), and we wanted to preserve the state-level nature of the BRFSS weighting process. Validation of the BRFSS SAEs will continue. The BRFSS will also be developing new methods for calculating standard errors for the SAEs, which will be comparable with direct estimates. The demand for substate health information is of paramount importance as states target communities to make the best use of resources. SAE is an invaluable tool for states and communities in this effort.

## Appendix Direct and BRFSS SAE Estimates for Health Status and Health Access Indicators for Counties That Had ≥500 Respondents in 2013

This file is available for download as a Microsoft Word document.
