# Supplementary material for: A Methodological Approach to Small Area Estimation for the Behavioral Risk Factor Surveillance System
Source: Prev Chronic Dis. 2016 Jul 14;13:E91. doi: 10.5888/pcd13.150480 (PMC4951081; doi:10.5888/pcd13.150480)
Supplement: Supplementary file 1 [file 15_0480_Appendix.docx]

| Table A-1. Direct and BRFSS SAE Estimates with Confidence Intervals^a^ for Health Status and Health Access Indicators  for Counties That Had ≥ 500 Respondents in 2013 | | | | | | | | | | | |
| --- | --- | --- | --- | --- | --- | --- | --- | --- | --- | --- | --- |
| State | County/ Borough/ Parish | Health Status / Access Indicator | | | | | | | | | |
|  |  | Direct fair/ poor health estimate | BRFSS SAE fair/ poor estimate | Direct physically unhealthy days estimate | BRFSS SAE physically unhealthy days estimate | Direct mentally unhealthy days estimate | BRFSS SAE mentally unhealthy days estimate | Direct delay due to medical cost estimate | BRFSS SAE delay due to medical cost estimate | Direct uninsured estimate | BRFSS SAE uninsured estimate |
| AL | Jefferson | 19.91  (16.65-23.65) | 21.43  (21.40-21.56) | 10.20  (7.88-13.12) | 12.48  (12.46-  12.50) | 13.12  (10.35-16.49) | 13.06  (13.04-13.09) | 13.42  (10.61-16.84) | 13.45  (13.42-13.47) | 19.46  (15.10-24.71) | 18.53  (18.50-18.56) |
| AK | Anchorage | 13.09  (10.62-16.00) | 13.33  (13.28-13.39) | 9.13  (6.99-11.85) | 9.44  (9.40-  9.49) | 7.59  (5.61-10.19) | 7.44  (7.41-7.48) | 14.88  (12.06-18.22) | 14.72  (14.67-14.78) | 18.21  (14.94-22.01) | 17.66  (17.60-17.72) |
| AK | Fairbanks North Star | 15.50  (12.22-19.50) | 15.88  (15.82-15.92) | 9.03  (6.67-12.11) | 9.74  (9.69-  9.79) | 8.37  (5.94-11.67) | 8.27  (8.22-8.32) | 13.55  (10.52-17.29) | 13.58  (13.52-13.64) | 16.22  (12.37-20.99) | 15.86  (15.80-15.93) |
| AK | Kenai Peninsula | 19.04  (15.42-23.30) | 17.12  (17.06-17.19) | 12.04  (9.22-15.56) | 10.94  (10.89-10.99) | 9.51  (6.91-12.96) | 9.10  (9.05-9.15) | 12.47  (9.43-16.32) | 12.92  (12.86-12.97) | 28.11  (23.01-33.85) | 27.54  (27.46-27.63) |
| AK | Matanuska-Susitna | 16.44  (12.79-20.9) | 16.3  (16.25-16.36) | 11.48  (8.67-15.05) | 10.98  (10.93-11.03) | 10.44  (7.56-14.26) | 9.97  (9.92-10.02) | 16.60  (12.97-21.00) | 16.27  (16.21-16.33) | 21.29  (16.81-26.58) | 20.46  (20.39-20.53) |
| AZ | Maricopa | 14.85  (11.93-18.30) | 15.55  (15.54-15.56) | 12.54  (10.14-15.42) | 12.99  (12.98-13.00) | 13.02  (10.18-16.50) | 12.58  (12.57-12.59) | 16.95  (13.74-20.73) | 17.00  (16.99-17.01) | 26.77  (22.35-31.71) | 25.29  (25.28-25.31) |
| AZ | Pima | 16.26  (12.85-20.40) | 16.77  (16.74-16.79) | 7.16  (5.26-9.27) | 8.79  (8.78-  8.81) | 10.02  (7.34-13.54) | 10.37  (10.35-10.38) | 16.22  (12.64-20.59) | 15.95  (15.93-15.97) | 19.86  (15.22-25.49) | 18.95  (18.93-18.98) |
| AZ | Pinal | 21.86  (17.54-26.90) | 23.52  (23.47-23.57) | 17.07  (13.37-21.53) | 18.16  (18.11-18.20) | 11.35  (8.11-15.67) | 10.92  (10.88-10.95) | 18.56  (13.70-24.65) | 17.25  (17.21-17.29) | 22.87  (16.45-30.87) | 21.31  (21.25-21.36) |
| AR | Pulaski | 23.86  (19.47-28.88) | 24.51  (24.47-24.56) | 13.01  (9.85-16.99) | 14.45  (14.41-14.49) | 13.52  (9.99-18.04) | 13.56  (13.52-13.60) | 21.03  (16.69-26.13) | 19.73  (19.68-19.77) | 25.60  (20.03-32.09) | 24.51  (24.45-24.56) |
| CA | Alameda | 13.36  (10.26-17.20) | 13.59  (13.58-13.61) | 8.62  (6.08-12.08) | 9.02  (9.01-  9.03) | 8.22  (5.60-11.91) | 8.81  (8.79-8.82) | 17.54  (13.13-23.05) | 15.70  (15.69-15.72) | 11.55  (8.39-15.69) | 11.33  (11.32-11.35) |
| CA | Los Angeles | 20.74  (18.59-23.10) | 20.26  (20.25-20.26) | 10.59  (8.98-12.46) | 10.67  (10.66-10.67) | 10.46  (8.86-12.30) | 10.69  (10.69-10.69) | 18.15  (16.08-20.43) | 17.40  (17.39-17.40) | 25.04  (22.38-27.89) | 24.29  (24.28-24.29) |
| CA | Orange | 16.41  (13.69-19.60) | 15.92  (15.91-15.92) | 8.87  (6.98-11.22) | 8.99  (8.98-  8.99) | 9.44  (6.78-12.99) | 9.65  (9.64-9.66) | 14.72  (11.88-18.09) | 14.05  (14.04-14.06) | 20.29  (16.27-25.01) | 18.88  (18.87-18.89) |
| CA | Riverside | 23.31  (11.51-27.60) | 22.31  (22.29-22.32) | 16.11  (12.69-20.24) | 16.17  (16.16-16.19) | 14.41  (11.11-18.48) | 13.69  (13.67-13.70) | 16.36  (13.26-20.02) | 14.97  (14.96-14.98) | 20.86  (16.57-25.92) | 19.08  (19.06-19.09) |
| CA | Sacramento | 21.52  (16.79-27.20) | 20.80  (20.79-20.82) | 10.32  (7.57-13.92) | 10.83  (10.82-10.85) | 16.74  (12.46-22.13) | 15.69  (15.68-15.71) | 14.81  (11.43-18.98) | 13.89  (13.88-13.90) | 21.97  (17.23-27.57) | 20.52  (20.50-20.54) |
| CA | San Bernardino | 19.7  (16.18-23.80) | 18.89  (18.87-18.90) | 12.73  (9.96-16.13) | 12.82  (12.80-12.83) | 11.14  (8.41-14.61) | 11.03  (11.02-11.04) | 16.08  (12.86-19.93) | 15.35  (15.33-15.36) | 21.59  (17.64-26.14) | 20.16  (20.14-20.18) |
| CA | San Diego | 15.15  (11.61-19.50) | 15.27  (15.26-15.28) | 6.80  (4.91-9.34) | 7.55  (7.55-  7.56) | 9.87  (7.38-13.08) | 10.11  (10.10-10.12) | 12.68  (9.55-16.64) | 12.91  (12.90-12.92) | 16.27  (12.36-21.11) | 15.38  (15.36-15.39) |
| CA | Santa Clara | 14.51  (10.84-19.20) | 14.25  (14.24-14.26) | 8.88  (6.35-12.29) | 9.24  (9.23-  9.25) | 7.68  (5.34-10.94) | 8.38  (8.37-8.39) | 12.12  (8.77-16.52) | 12.31  (12.30-12.32) | 18.11  (13.55-23.79) | 17.05  (17.03-17.06) |
| CO | Adams | 19.04  (16.07-22.40) | 17.20  (17.18-17.21) | 9.71  (7.72-12.15) | 9.66  (9.65-  9.67) | 9.28  (7.20-11.89) | 9.08  (9.06-9.09) | 18.28  (15.33-21.64) | 16.27  (16.25-16.28) | 30.45  (26.24-35.03) | 25.41  (25.39-25.43) |
| CO | Arapahoe | 10.9  (8.87-13.30) | 10.76  (10.75-10.78) | 7.38  (5.70-9.52) | 7.42  (7.41-  7.43) | 8.50  (6.67-10.78) | 8.38  (8.37-8.39) | 15.68  (13.17-18.57) | 14.92  (14.9-14.93) | 18.43  (15.39-21.92) | 17.60  (17.58-17.62) |
| CO | Boulder | 11.08  (8.34-14.60) | 11.18  (11.16-11.21) | 9.03  (6.67-12.12) | 9.09  (9.07-  9.11) | 7.01  (4.82-10.09) | 7.25  (7.23-7.27) | 10.78  (7.93-14.49) | 10.80  (10.78-10.82) | 15.34  (11.50-20.17) | 14.06  (14.03-14.09) |
| CO | Denver | 15.85  (13.60-18.40) | 15.67  (15.65-15.69) | 8.02  (6.49-9.88) | 8.13  (8.12-  8.15) | 8.94  (7.25-10.99) | 8.97  (8.95-8.98) | 18.70  (16.25-21.42) | 17.78  (17.76-17.81) | 24.14  (21.10-27.46) | 22.31  (22.29-22.34) |
| CO | Douglas | 7.84  (5.76-10.60) | 8.39  (8.36-8.41) | 5.78  (3.96-8.35) | 6.47  (6.45-  6.49) | 7.01  (4.92-9.89) | 7.33  (7.31-7.35) | 6.73  (4.61-9.73) | 7.45  (7.43-  7.47) | 5.11  (3.17-8.12) | 5.70  (5.68-5.73) |
| CO | El Paso | 12.49  (10.45-14.90) | 12.24  (12.22-12.26) | 11.09  (9.11-13.44) | 11.06  (11.04-11.08) | 10.80  (8.73-13.28) | 10.51  (10.49-10.52) | 14.19  (11.75-17.03) | 13.29  (13.28-13.31) | 16.98  (14.12-20.29) | 15.56  (15.54-15.58) |
| CO | Jefferson | 10.37  (8.62-12.40) | 10.31  (10.30-10.32 | 8.16  (6.58-10.09) | 8.12  (8.11-  8.13) | 8.23  (6.52-10.34) | 8.30  (8.29-8.31) | 11.37  (9.26-13.88) | 11.35  (11.34-11.36) | 12.87  (10.30-15.97) | 12.04  (12.02-12.05) |
| CO | Larimer | 8.77  (6.58-11.60) | 9.10  (9.08-9.13) | 6.08  (4.36-8.42) | 6.56  (6.54-  6.58) | 8.67  (6.47-11.54) | 8.69  (8.67-8.72) | 13.16  (9.64-17.71) | 13.05  (13.02-13.08) | 16.88 (12.47-22.45) | 15.68  (15.64-15.71) |
| CO | Mesa | 13.08  (10.09-16.80) | 13.43  (13.40-13.47) | 11.85  (8.93-15.57) | 11.75  (11.72-11.78) | 12.45  (9.17-16.69) | 11.64  (11.61-11.67) | 16.26  (12.49-20.91) | 15.37  (15.33-15.40) | 25.45  (19.90-31.92) | 24.46  (24.41-24.51) |
| CO | Weld | 12.44  (9.63-15.90) | 11.97  (11.95-11.99) | 9.35  (7.00-12.38) | 9.38  (9.36-  9.40) | 7.10  (5.05-9.90) | 7.24  (7.23-7.26) | 15.44  (12.11-19.49) | 14.14  (14.12-14.16) | 18.72  (14.65-23.63) | 16.83  (16.81-16.86) |
| CT | Fairfield | 13.38  (11.28-15.80) | 12.90  (12.88-12.92) | 8.80  (7.10-10.85) | 8.68  (8.66-  8.69) | 7.82  (6.28-9.71) | 7.94  (7.93-7.95) | 12.85  (10.78-15.25) | 11.65  (11.64-11.66) | 13.32  (10.88-16.20) | 11.45  (11.43-11.47) |
| CT | Hartford | 13.79  (11.82-16.00) | 13.36  (13.34-13.37) | 9.35  (7.55-11.53 | 9.23  (9.22-  9.24) | 9.65 (7.90-11.72) | 9.69  (9.68-9.71) | 11.83  (9.92-14.06) | 11.54  (11.52-11.55) | 12.83  (10.38-15.76) | 11.21  (11.19-11.22) |
| CT | New Haven | 13.81  (11.47-16.50) | 13.39  (13.37-13.41) | 9.28  (7.53-11.38 | 9.28  (9.26-  9.29) | 12.06  (9.89-14.64) | 11.42  (11.40-11.43) | 13.31  (10.90-16.16) | 11.40  (11.39-11.42) | 14.18  (11.23-17.76) | 12.29  (12.27-12.31) |
| CT | New London | 12.39  (9.11-16.60) | 12.19  (12.16-12.22) | 6.66  (4.64-9.49) | 7.08  (7.05-  7.10) | 12.23  (8.74-16.85) | 10.96  (10.93-10.99) | 12.07  (8.71-16.50) | 10.11  (10.09-10.14) | 6.78  (4.19-10.79) | 7.13  (7.11-7.16) |
| CT | Tolland | 7.91  (5.81-10.70) | 8.63  (8.60-8.66) | 5.76  (3.83-8.56) | 6.12  (6.10-  6.15) | 8.54  (5.75-12.50) | 8.73  (8.70-  8.76 | 9.05  (6.48-12.50) | 9.66  (9.63-  9.68) | 7.08  (4.53-10.90) | 7.17  (7.14-7.21) |
| CT | Windham | 16.03  (12.77-19.90) | 14.98  (14.93-15.03) | 13.76  (10.62-17.65) | 12.96  (12.91-13.01) | 10.38  (7.62-14.01) | 10.23  (10.19-10.27) | 9.95  (7.46-13.16) | 10.14  (10.11-10.17) | 12.51  (9.48-16.32) | 11.00  (10.95-11.05) |
| DE | Kent | 17.95  (15.17-21.11) | 17.26  (17.21-17.30) | 10.78  (8.53-13.54) | 11.09  (11.05-11.13) | 10.84  (8.42-13.84) | 10.71  (10.67-10.74) | 13.85  (11.22-16.99) | 12.85  (12.81-12.90) | 14.25  (11.21-17.95) | 12.75  (12.70-12.80) |
| DE | New Castle | 17.04  (15.22-19.03) | 17.17  (17.15-17.20) | 10.46  (9.05-12.06) | 10.99  (10.97-11.01) | 10.71  (9.25-12.38) | 10.70  (10.68-10.72) | 11.43  (9.77-13.32) | 10.92  (10.90-10.95) | 13.18  (11.19-15.46) | 12.09  (12.07-12.12) |
| DE | Sussex | 17.55  (15.03-20.04) | 18.21  (18.17-18.24) | 12.07  (9.92-14.62) | 11.81  (11.78-11.83) | 10.34  (8.09-13.13) | 9.96  (9.93-9.99) | 13.37  (11.08-16.05) | 12.05  (12.02-12.08) | 17.80  (14.60-21.53) | 15.58  (15.53-15.63) |
| DC | District of Columbia | 12.72  (11.35-14.23) | 13.32  (13.30-13.34) | 7.94  (6.80-9.24) | 8.37  (8.35-  8.39) | 9.28  (7.94-10.82) | 9.00  (8.99-9.02) | 10.75  (9.27-12.44) | 10.43  (10.41-10.45) | 9.85  (8.16-11.85) | 9.35  (9.33-9.37) |
| FL | Alachua | 11.46  (8.60-15.10) | 12.81  (12.79-12.84) | 7.97  (5.69-11.05) | 9.37  (9.35-  9.39) | 10.41  (7.48-14.31) | 10.25  (10.23-10.28) | 19.51  (15.13-24.78) | 18.93  (18.90-18.96) | 22.95  (17.75-29.12) | 23.39 (23.35-23.43) |
| FL | Bay | 20.11  (16.50-24.30) | 20.67  (20.64-20.70) | 13.19  (10.15-16.96) | 13.43  (13.40-13.45) | 15.05  (11.71-19.12) | 14.38  (14.35-14.40) | 21.33  (17.40-25.88) | 19.65  (19.62-19.67) | 28.80  (23.48-34.77) | 27.29  (27.25-27.33) |
| FL | Brevard | 21.80  (17.43-26.90) | 21.56  (21.53-21.58) | 14.36  (11.13-18.34) | 14.31  (14.29-14.33) | 10.65  (7.70-14.57) | 10.67  (10.66-10.69) | 15.65 (11.98-20.19) | 15.30  (15.28-15.32) | 30.62  (24.02-38.13) | 28.40  (28.37-28.44) |
| FL | Broward | 18.01  (14.11-22.70) | 18.18  (18.17-18.20) | 13.68  (9.98-18.47) | 13.96  (13.95-13.97) | 13.44  (9.95-17.90) | 13.17  (13.16-13.18) | 24.52  (19.86-29.87) | 22.84  (22.83-22.86) | 26.60  (21.06-32.98) | 24.63  (24.62-24.65) |
| FL | Collier | 17.01  (12.69-22.40) | 17.64  (17.61-17.67) | 12.17  (8.56-17.00) | 13.07  (13.04-13.09) | 7.55  (4.99-11.28) | 8.07  (8.04-8.09) | 18.01  (13.41-23.75) | 16.58  (16.55-16.61) | 38.55  (30.56-47.21) | 34.08  (34.02-34.14) |
| FL | Duval | 19.87  (17.08-23.00) | 20.28  (20.26-20.30) | 12.55  (10.38-15.08) | 13.13  (13.12-13.14) | 13.05  (10.69-15.84) | 12.74  (12.72-12.75) | 20.48  (17.51-23.80) | 19.56  (19.55-19.58) | 23.56  (19.95-27.60) | 22.66  (22.64-22.68) |
| FL | Escambia | 18.90  (15.85-22.40) | 19.84  (19.81-19.87) | 14.48  (11.56-17.99) | 14.40  (14.38-14.43) | 10.57  (8.15-13.61) | 10.55  (10.53-10.57) | 17.53  (14.34-21.26) | 16.79  (16.77-16.82) | 25.62  (21.24-30.54) | 24.78  (24.75-24.82) |
| FL | Hills | 19.61  (16.24-23.50) | 19.64  (19.62-19.65) | 11.36  (8.75-14.62) | 12.16  (12.15-12.17) | 12.84  (9.99-16.37) | 12.41  (12.40-12.42) | 17.66  (14.34-21.54) | 17.13  (17.12-17.15) | 22.91  (18.67-27.78) | 22.20  (22.18-22.22) |
| FL | Lake | 20.62  (15.92-26.30) | 21.41  (21.38-21.44) | 12.90  (9.62-17.08) | 14.37  (14.35-14.39) | 10.18  (7.17-14.28) | 10.05  (10.03-10.07) | 20.28  (15.35-26.30) | 17.52  (17.50-17.55) | 29.42  (22.03-38.07) | 26.24  (26.20-26.28) |
| FL | Lee | 19.07  (15.89-22.70) | 19.33  (19.32-19.35) | 14.38  (11.51-17.83) | 14.50  (14.48-14.52) | 9.57  (7.15-12.70) | 9.60  (9.59-9.62) | 19.87  (16.60-23.59) | 18.33  (18.31-18.35) | 34.70  (29.41-40.41) | 31.65  (31.62-31.68) |
| FL | Leon | 10.76  (7.99-14.30) | 13.15  (13.12-13.18) | 10.87  (7.41-15.66) | 11.73  (11.71-11.76) | 7.48  (5.24-10.58) | 8.66  (8.64-8.68) | 12.81  (9.17-17.61) | 13.64  (13.62-13.67) | 18.74  (13.48-25.45) | 18.91  (18.88-18.94) |
| FL | Levy | 31.28  (24.86-38.50) | 28.21  (28.15-28.26) | 18.64  (14.56-23.54) | 17.43  (17.38-17.47) | 18.42  (12.74-25.87) | 17.22  (17.17-17.27) | 24.40  (18.23-31.85) | 23.38  (23.33-23.43) | 32.24  (23.83-41.97) | 30.98  (30.91-31.06) |
| FL | Manatee | 21.20  (16.83-26.40) | 21.16  (21.13-21.19) | 16.08  (12.43-20.55) | 15.72  (15.70-15.75) | 10.85  (7.90-14.72) | 10.68  (10.66-10.71) | 19.41  (15.08-24.61) | 18.05  (18.02-18.08) | 30.36  (23.94-37.66) | 27.83  (27.79-27.88) |
| FL | Marion | 19.59  (15.96-23.80) | 20.19  (20.16-20.22) | 11.43  (8.79-14.73) | 12.42  (12.40-12.45) | 11.39  (8.56-15.00) | 11.09  (11.07-11.12) | 20.98  (16.93-25.71) | 19.26  (19.23-19.29) | 31.60  (25.58-38.30) | 28.69  (28.65-28.74) |
| FL | Miami-Dade | 20.86  (16.93-25.40) | 21.57  (21.56-21.58) | 13.86  (10.6-17.92) | 14.72  (14.71-14.73) | 13.83  (10.31-18.29) | 12.98  (12.97-12.99) | 24.37  (20.19-29.10) | 23.29  (23.28-23.30) | 38.84  (33.20-44.80) | 37.72  (37.70-37.73) |
| FL | Okaloosa | 18.28  (14.72-22.50) | 18.41  (18.37-18.45) | 13.73  (10.66-17.51) | 13.92  (13.89-13.96) | 10.70  (7.98-14.21) | 10.99  (10.96-11.02) | 17.94  (14.22-22.39) | 17.07  (17.03-17.11) | 21.10  (16.55-26.52) | 20.52  (20.48-20.57) |
| FL | Orange | 19.58  (16.05-23.70) | 19.62  (19.61-19.64) | 12.39  (9.55-15.93) | 12.86  (12.85-12.88) | 9.99  (7.32-13.48) | 10.38  (10.37-10.39) | 24.50  (20.49-29.01) | 23.27  (23.25-23.28) | 30.96  (26.11-36.26) | 29.23  (29.21-29.25) |
| FL | Palm Beach | 16.52  (12.46-21.60) | 17.55  (17.54-17.57) | 10.32  (7.33-14.35) | 11.58  (11.57-11.59) | 8.98  (5.97-13.31) | 9.83  (9.82-9.85) | 23.02  (17.59-29.54) | 21.69  (21.68-21.71) | 26.99  (20.32-34.88) | 25.66  (25.64-25.68) |
| FL | Pasco | 18.67  (14.70-23.40) | 19.35  (19.32-19.37) | 12.96  (9.73-17.07) | 13.82  (13.80-13.85) | 14.85  (11.06-19.66) | 13.83  (13.80-13.85) | 15.60  (11.82-20.31) | 15.13  (15.11-15.15) | 22.28  (16.44-29.47) | 21.74  (21.71-21.78) |
| FL | Pinellas | 18.68  (14.94-23.10) | 19.17  (19.15-19.19) | 12.70  (9.52-16.74) | 13.00  (12.99-13.02) | 10.94  (8.02-14.75) | 10.85  (10.83-10.86) | 16.22  (12.64-20.56) | 15.70  (15.69-15.72) | 25.53  (20.17-31.75) | 24.33  (24.31-24.36) |
| FL | Polk | 24.87  (20.01-30.50) | 24.82  (24.79-24.84) | 16.77  (12.41-22.26) | 16.92  (16.90-16.94) | 13.04  (9.06-18.42) | 12.58  (12.56-12.60) | 20.41  (15.83-25.92) | 19.17  (19.15-19.20) | 30.53  (23.74-38.29) | 28.16 (28.13-28.19) |
| FL | St. Lucie | 21.39  (16.46-27.30) | 22.05  (22.01-22.09) | 13.57  (9.91-18.32) | 14.21  (14.18-14.24) | 13.83  (10.13-18.6) | 13.02  (12.99-13.05) | 23.87  (18.92-29.66) | 22.06  (22.03-22.10) | 32.69  (25.58-40.70) | 31.25  (31.19-31.30) |
| FL | Santa Rosa | 17.06  (13.25-21.70) | 17.47  (17.42-17.51) | 12.36  (9.37-16.13) | 12.82  (12.78-12.85) | 10.83  (7.79-14.88) | 10.97  (10.94-11.01) | 14.56  (11.22-18.68) | 14.70  (14.66-14.74) | 25.00  (19.72-31.14) | 23.89  (23.83-23.95) |
| FL | Sarasota | 18.60  (15.02-22.80) | 19.13  (19.11-19.16) | 14.22  (11.15-17.97) | 14.40  (14.38-14.42) | 13.14  (9.94-17.18) | 12.95  (12.93-12.98) | 20.08  (16.34-24.43) | 19.36  (19.34-19.39) | 32.28  (26.08-39.17) | 30.15  (30.10-30.20) |
| FL | Seminole | 19.47  (14.57-25.50) | 19.89  (19.87-19.92) | 11.63  (7.95-16.69) | 12.71  (12.69-12.73) | 11.76  (8.31-16.38) | 11.53  (11.51-11.55) | 19.15  (14.25-25.24) | 18.65  (18.62-18.67) | 25.89  (19.27-33.83) | 24.16  (24.13-24.19) |
| FL | Volusia | 17.15  (13.87-21.00) | 17.86  (17.84-17.88) | 13.26  (10.30-16.90) | 14.09  (14.07-14.11) | 13.30  (10.10-17.33) | 12.83  (12.81-12.84) | 21.57  (17.49-26.31) | 19.92  (19.89-19.94) | 26.79  (21.29-33.11) | 25.12  (25.09-25.16) |
| GA | Fulton | 14.31  (11.29-18.00) | 14.79  (14.77-14.81) | 8.06  (5.95-10.84) | 8.88  (8.87-  8.90) | 6.51  (4.46-9.43) | 6.82  (6.81-6.84) | 17.04  (13.81-20.83) | 16.37  (16.35-16.40) | 24.57  (19.87-29.98) | 23.07  (23.04-23.10) |
| HI | Hawaii | 15.04  (12.70-17.70) | 15.26  (15.22-15.29) | 10.78  (8.77-13.18) | 10.65  (10.62-10.69) | 8.95  (7.26-10.98 | 8.73  (8.70-8.76) | 12.83  (10.45-15.66) | 12.32  (12.29-12.36) | 13.01  (10.61-15.87) | 12.47  (12.43-12.51) |
| HI | Honolulu | 13.43  (12.09-14.90) | 14.12  (14.10-14.13) | 7.15  (6.15-8.29) | 7.44  (7.43-  7.45) | 7.25  (6.29-8.34) | 7.03  (7.02-7.04) | 7.12  (6.18-8.19) | 6.94  (6.93-  6.95) | 8.72  (7.51-10.10) | 8.59  (8.58-8.60) |
| HI | Kauai | 12.85  (10.42-15.70) | 14.35  (14.32-14.38) | 7.92  (5.81-10.69) | 8.50  (8.47-  8.53) | 5.08  (3.72-6.90) | 5.75  (5.73-5.77) | 11.19  (8.65-14.37) | 10.97  (10.93-11.00) | 15.59  (11.66-20.54) | 14.70 (14.66-14.75) |
| HI | Maui | 14.79  (12.03-18.10) | 14.35  (14.32-14.38) | 9.73  (7.51-12.51) | 9.83  (9.80-  9.86) | 8.48  (6.68-10.71) | 8.16  (8.13-8.18) | 11.50  (9.23-14.24) | 11.04  (11.01-11.08) | 11.90  (9.56-14.73) | 11.59  (11.56-11.63) |
| ID | Ada | 8.76  (6.50-11.70) | 9.46  (9.44-9.48) | 5.81  (4.20-8.00) | 6.60  (6.58-  6.61) | 9.20  (6.82-12.30) | 9.51  (9.47-9.55) | 16.75  (13.46-20.64) | 16.25  (16.22-16.28) | 20.10  (16.10-24.80) | 20.22 (20.19-20.26) |
| ID | Canyon | 17.93  (13.62-23.20) | 18.45  (18.41-18.50) | 11.00  (8.09-14.77) | 11.71  (11.67-11.75) | 12.61  (9.26-16.95) | 9.73  (9.70-9.76) | 18.40  (13.92-23.93) | 17.24  (17.19-17.28) | 29.32  (22.99-36.57) | 25.85  (25.79-25.91) |
| IL | Cook | 16.96  (14.57-19.70) | 16.81  (16.80-16.82) | 8.72  (7.01-10.80) | 9.05  (9.05-  9.06) | 12.05  (9.90-14.60) | 9.11  (9.09-9.13) | 15.92  (13.27-18.99) | 14.95  (14.94-14.96) | 22.53  (19.03-26.45) | 20.62 (20.60-20.63) |
| IN | Allen | 13.29  (10.27-17.00) | 14.44  (14.41-14.47) | 10.11  (7.59-13.34) | 10.82  (10.80-10.85) | 9.16  (6.49-12.79) | 11.91  (11.87-11.94) | 15.42  (11.86-19.80) | 15.08  (15.04-15.11) | 23.16  (18.34-28.80) | 21.21  (21.17-21.25) |
| IN | Lake | 22.36  (18.30-27.00) | 23.16  (23.12-23.19) | 16.33  (12.67-20.79) | 15.17  (15.14-15.20) | 13.52  (9.97-18.07) | 11.80  (11.79-11.81) | 18.86  (14.84-23.66) | 17.97 (17.94-18.01) | 19.93  (15.06-25.89) | 18.71  (18.67-18.75) |
| IN | Marion | 17.94  (15.34-20.90) | 18.24  (18.22-18.26) | 9.97  (8.18-12.08) | 10.80  (10.78-10.81) | 10.48  (8.41-12.99) | 9.57  (9.54-9.59) | 17.87  (15.05-21.08) | 16.72  (16.70-16.74) | 28.08  (24.31-32.18) | 25.81 (25.79-25.84) |
| IA | Linn | 14.92  (10.96-20.00) | 14.83  (14.78-14.88) | 8.37  (5.69-12.16) | 8.53  (8.49-  8.57) | 9.44  (6.13-14.28) | 13.02  (12.99-13.05) | 7.52  (4.66-11.93) | 7.61  (7.58-  7.65) | 14.76  (10.45-20.45) | 13.84  (13.79-13.90) |
| IA | Polk | 12.36  (9.97-15.20) | 12.39  (12.36-12.42) | 8.20  (6.25-10.70) | 8.29  (8.27-  8.32) | 10.12  (7.73-13.14) | 10.38  (10.37-10.40) | 10.75  (8.19-14.00) | 10.38  (10.35-10.41) | 11.50  (8.37-15.61) | 11.39  (11.35-11.42) |
| KS | Butler | 17.07  (13.55-21.30) | 17.95  (17.92-17.98) | 10.11  (7.45-13.58) | 10.40  (10.38-10.43) | 7.39  (4.99-10.82) | 8.00  (7.98-8.02) | 14.58  (11.06-18.99) | 14.96  (14.93-14.99) | 20.20  (15.51-25.87) | 21.63  (21.59-21.67) |
| KS | Douglas | 10.35  (8.04-13.20) | 10.65  (10.55-10.75) | 6.17  (4.70-8.07) | 6.56  (6.49-  6.64) | 9.38  (7.05-12.38) | 9.49  (9.40-9.58) | 13.58  (10.89-16.80) | 13.50  (13.38-13.61) | 16.03  (12.61-20.17) | 16.32  (16.18-16.45) |
| KS | Johnson | 8.96  (7.93-10.10) | 9.21  (9.20-9.22) | 6.35  (5.50-7.31) | 6.72  (6.71-  6.73) | 6.39  (5.49-7.42) | 6.57  (6.56-6.58) | 9.93  (8.77-11.23) | 9.75  (9.74-  9.76) | 12.73  (11.09-14.57) | 12.15  (12.14-12.17) |
| KS | Leavenworth | 18.49  (14.54-23.20) | 18.56  (18.44-18.68) | 10.76  (7.93-14.45) | 11.29  (11.20-11.39) | 10.04  (6.91-14.36) | 9.78  (9.69-9.86) | 13.22  (9.84-17.54) | 11.95  (11.86-12.05) | 13.48  (9.25-19.24) | 12.19  (12.08-12.31) |
| KS | Reno | 14.27  (11.48-17.6) | 14.77  (14.72-14.81) | 11.51  (8.86-14.82) | 11.85  (11.80-11.89) | 9.18  (6.88-12.15) | 9.08  (9.05-9.12) | 13.59  (10.37-17.60) | 12.70  (12.66-12.75) | 20.58  (15.65-26.57) | 18.50  (18.44-18.57) |
| KS | Sedgwick | 16.10  (14.71-17.60) | 16.12  (16.11-16.14) | 10.07  (8.97-11.29) | 10.54  (10.53-10.55) | 10.31  (9.09-11.68) | 10.24  (10.23-10.26) | 16.33  (14.73-18.07) | 15.20  (15.19-15.22) | 24.06  (21.91-26.35) | 21.52  (21.50-21.54) |
| KS | Shawnee | 18.92  (16.82-21.20) | 18.79  (18.77-18.82) | 10.56  (9.01-12.33) | 11.08  (11.06-11.10) | 10.25  (8.58-12.21) | 9.97  (9.95-9.98) | 15.72  (13.61-18.09) | 14.36  (14.34-14.38) | 18.56  (15.88-21.57) | 16.24  (16.22-16.27 |
| KS | Wyandotte | 23.04  (20.18-26.20) | 22.98  (22.93-23.03) | 12.44  (10.15-15.15) | 12.79  (12.76-12.83) | 11.98  (9.88-14.45) | 11.91  (11.87-11.94) | 22.14  (19.10-25.50) | 20.67  (20.62-20.71) | 38.42  (34.27-42.73) | 35.42  (35.36-35.48) |
| KY | Jefferson | 20.77  (17.50-24.50) | 22.05  (22.03-22.07) | 10.62  (8.36-13.41) | 11.55  (11.53-11.56) | 11.86  (9.42-14.83) | 11.85  (11.84-11.87) | 17.75  (14.58-21.44) | 17.21  (17.19-17.23) | 19.39  (15.46-24.03) | 18.30  (18.28-18.32) |
| LA | East Baton Rouge | 15.97  (11.82-21.20) | 18.09  (18.05-18.13) | 6.03  (4.30-8.39) | 7.02  (6.99-  7.04) | 8.24  (5.41-12.34) | 8.31  (8.28-8.34) | 16.28  (11.78-22.07) | 15.99  (15.95-16.03) | 21.24  (14.63-29.79) | 20.61  (20.56-20.66) |
| ME | Androscoggin | 17.27  (13.54-21.80) | 17.82  (17.75-17.90) | 13.77  (10.51-17.84) | 13.37  (13.31-13.43) | 10.70  (7.87-14.41) | 11.27  (11.22-11.32) | 9.89  (6.73-14.33) | 10.36  (10.31-10.41) | 14.52  (10.19-20.28) | 14.87  (14.80-14.95) |
| ME | Cumberland | 10.23  (8.34-12.50) | 10.91  (10.89-10.93) | 8.81  (7.03-10.99) | 9.49  (9.47-  9.51) | 11.08  (8.85-13.79) | 10.72  (10.70-10.74) | 9.29  (7.24-11.85) | 9.29  (9.27-  9.31) | 12.39  (9.62-15.82) | 12.48 (12.45-12.50) |
| ME | Kennebec | 14.30  (11.45-17.70) | 14.84  (14.79-14.89) | 11.41  (8.83-14.63) | 11.79  (11.74-11.83) | 9.94  (7.29-13.42) | 10.48  (10.45-10.52) | 9.12  (6.67-12.35) | 9.50  (9.46-  9.53) | 14.55  (10.69-19.50) | 14.31  (14.25-14.37) |
| ME | Penobscot | 18.51  (15.29-22.20) | 19.08  (18.98-19.19) | 12.75  (10.09-15.99) | 13.44  (13.35-13.52) | 12.47  (9.72-15.88) | 11.44  (11.37-11.52) | 9.86  (7.28-13.23) | 9.67  (9.60-  9.74) | 14.29  (11.00-18.36) | 14.02  (13.91-14.12) |
| ME | York | 12.75  (10.44-15.50) | 13.38  (13.35-13.41) | 11.85  (9.59-14.55) | 11.92  (11.89-11.95) | 13.75  (11.09-16.93) | 12.05  (12.02-12.08) | 10.46  (8.22-13.22) | 10.33  (10.30-10.36) | 14.79  (11.78-18.41) | 14.95  (14.91-14.99) |
| MD | Anne Arundel | 13.26  (10.63-16.40) | 13.37  (13.35-13.40) | 9.12  (6.92-11.94) | 9.27  (9.25-  9.29) | 9.89  (7.21-13.42) | 9.77  (9.75-9.79) | 11.03  (8.33-14.47) | 10.76  (10.74-10.78) | 9.94  (6.77-14.36) | 9.71  (9.69-9.73) |
| MD | Baltimore | 15.32  (12.91-18.10) | 15.14  (15.13-15.16) | 10.20  (8.39-12.35) | 10.55  (10.54-10.56) | 8.94  (7.09-11.21) | 8.93  (8.92-8.94) | 13.18  (10.79-16.01) | 12.13  (12.11-12.14) | 16.22  (12.88-20.22) | 14.26  (14.24-14.28) |
| MD | Charles | 14.26  (10.90-18.40) | 14.32  (14.27-14.36) | 9.65  (6.49-14.12) | 9.78  (9.74-  9.82) | 8.94  (6.22-12.69) | 8.94  (8.90-8.97) | 7.24  (4.91-10.57) | 7.21  (7.17-  7.24) | 6.07  (3.67-9.87) | 5.95  (5.92-5.99) |
| MD | Frederick | 10.17  (7.67-13.40) | 10.64  (10.61-10.67) | 4.77  (3.31-6.82) | 5.74  (5.72-  5.76 | 7.20  (5.06-10.14) | 7.77  (7.75-7.80) | 4.25  (2.63-6.78) | 4.76  (4.75-  4.78) | 6.10  (3.66-9.99) | 6.07 (6.04-6.10) |
| MD | Montgomery | 12.03  (9.82-14.70) | 11.77  (11.76-11.79) | 6.89  (5.22-9.03) | 7.34  (7.33-  7.35) | 7.80  (5.93-10.19) | 7.88  (7.87-7.89) | 12.86  (10.50-15.65) | 11.43  (11.42-11.44) | 16.66  (13.68-20.13) | 13.39  (13.37-13.40) |
| MD | Prince George´s | 16.42  (13.54-19.80) | 16.43  (16.41-16.44) | 9.21  (6.95-12.10) | 9.52  (9.51-  9.53) | 8.55  (6.55-11.09) | 8.61  (8.60-8.62) | 18.09  (14.93-21.73) | 17.11  (17.09-17.13) | 25.54  (21.44-30.11) | 22.63  (22.61-22.65) |
| MD | Washington | 15.80  (11.83-20.80) | 16.07  (16.02-16.11) | 10.82  (7.09-16.17) | 11.22  (11.18-11.26) | 7.75  (5.37-11.05) | 8.53  (8.49-8.56) | 11.84  (8.08-17.03) | 11.28  (11.24-11.33) | 13.65  (8.83-20.51) | 12.97  (12.92-13.03) |
| MD | Baltimore city | 21.28  (17.43-25.70) | 20.64  (20.61-20.66) | 10.13  (7.42-13.68) | 10.18  (10.17-10.20) | 12.89  (9.67-16.99) | 12.16  (12.14-12.18) | 17.12  (13.48-21.50) | 15.75  (15.73-15.77) | 19.90  (15.07-25.81) | 17.27  (17.25-17.30) |
| MA | Bristol | 17.81  (14.59-21.60) | 17.26  (17.24-17.27) | 14.15  (11.02-17.98) | 14.34  (14.33-14.36) | 14.72  (11.56-18.57) | 13.90  (13.88-13.92) | 10.05  (7.64-13.11) | 9.59  (9.57-  9.60) | 7.47  (5.15-10.72) | 6.80  (6.79-6.82) |
| MA | Essex | 15.39  (12.95-18.20) | 13.38  (13.37-13.39) | 9.09  (7.32-11.23) | 8.90  (8.89-  8.91) | 10.63  (8.35-13.45) | 10.21  (10.20-10.22) | 10.35  (8.15-13.07) | 8.80  (8.79-  8.81) | 8.31  (5.99-11.43) | 6.34  (6.33-6.35) |
| MA | Hampden | 19.33  (16.07-23.10) | 17.15  (17.13-17.17) | 13.44  (10.47-17.08) | 12.22  (12.20-12.24) | 13.89  (10.74-17.78) | 12.95  (12.93-12.96) | 9.64  (7.22-12.75) | 8.59  (8.58-  8.61) | 8.53  (5.78-12.42) | 7.50 (7.48-7.51) |
| MA | Middlesex | 11.12  (9.60-12.90) | 11.35  (11.34-11.35) | 7.85  (6.55-9.38) | 8.19  (8.18-  8.19 | 9.52  (7.98-11.34) | 9.76  (9.75-9.77) | 6.48  (5.29-7.92) | 6.32  (6.32-  6.33) | 5.30 (3.97-7.04) | 5.41  (5.41-5.42) |
| MA | Norfolk | 11.56  (8.86-14.90) | 11.59  (11.57-11.60) | 9.19  (6.94-12.08) | 9.32  (9.31-  9.33) | 10.84  (8.04-14.47) | 10.74  (10.73-10.75) | 7.49  (5.36-10.37) | 7.35  (7.34-  7.36) | 5.86  (3.75-9.03) | 5.71  (5.70-5.72) |
| MA | Plymouth | 13.91  (10.98-17.47) | 13.50  (13.49-13.52) | 8.89  (6.68-11.75) | 8.99  (8.98-  9.00) | 11.99  (9.05-15.72) | 11.61  (11.59-11.62) | 10.55  (7.63-14.41) | 9.82  (9.81-  9.84) | 7.20  (4.48-11.38) | 6.28  (6.26-6.29) |
| MA | Suffolk | 13.99  (11.85-16.84) | 12.90  (12.88-12.91) | 9.97  (8.06-12.27) | 9.48  (9.46-  9.49) | 11.56  (9.44-14.08) | 11.11  (11.09-11.12) | 8.33  (6.65-10.40) | 7.50  (7.49-  7.51) | 10.39  (7.85-13.64) | 8.54  (8.53-8.56) |
| MA | Worcester | 15.13  (12.48-17.76) | 14.23  (14.22-14.24) | 10.03  (8.26-12.14) | 10.33  (10.32-10.34) | 11.31  (9.20-13.82) | 11.02  (11.01-11.03) | 9.77  (7.83-12.13) | 8.91  (8.90-  8.92) | 7.49  (5.51-10.12) | 6.19  (6.19-6.20) |
| MI | Kent | 13.43  (10.65-16.80) | 13.62  (13.60-13.64) | 10.33  (7.82-13.51) | 10.57  (10.55-10.59) | 10.24  (7.61-13.65) | 10.39  (10.37-10.41) | 16.02  (12.86-19.79) | 15.75  (15.72-15.77) | 18.33  (14.24-23.27) | 18.12  (18.09-18.15) |
| MI | Macomb | 18.44  (15.33-22.00) | 18.10  (18.08-18.13) | 10.69  (8.46-13.42) | 10.83  (10.81-10.85) | 10.72  (8.26-13.82) | 10.59  (10.57-10.61) | 16.09  (12.77-20.07) | 15.41  (15.39-15.44) | 20.45  (15.97-25.79) | 19.00  (18.97-19.03) |
| MI | Oakland | 12.79  (10.26-15.80) | 13.04  (13.02-13.05) | 8.15  (6.32-10.44 | 8.90  (8.89-  8.91) | 7.30  (5.58-9.49) | 8.29  (8.28-8.30) | 12.94  (10.25-16.21) | 12.77  (12.75-12.78) | 14.17  (10.79-18.40) | 13.27  (13.25-13.29) |
| MI | Wayne | 24.92  (22.19-27.90) | 25.05  (25.03-25.06) | 15.90  (13.48-18.66) | 15.74  (15.73-15.76) | 14.38  (12.12-16.98) | 13.85  (13.84-13.86) | 19.31  (16.69-22.23) | 18.50  (18.48-18.51) | 21.09  (18.04-24.51) | 19.69  (19.67-19.70) |
| MN | Anoka | 9.63  (6.55-13.90) | 9.49  (9.46-9.52) | 5.84  (3.55-9.47) | 5.68  (5.66-  5.70) | 7.88  (5.30-11.57) | 7.74  (7.71-7.76) | 13.24  (9.44-18.26) | 12.57  (12.54-12.61) | 11.74  (8.13-16.67) | 11.71 (11.67-11.75) |
| MN | Dakota | 13.76  (9.66-19.20) | 13.25  (13.21-13.28) | 8.35  (5.34-12.82) | 8.29  (8.26-  8.32) | 6.40  (4.24-9.57) | 6.36  (6.34-6.38) | 5.92  (4.04-8.61) | 6.00  (5.98-  6.02) | 7.01  (4.42-10.97) | 6.70  (6.68-6.73) |
| MN | Hennepin | 11.71  (9.77-14.00) | 11.17  (11.16-11.19) | 7.15  (5.67-8.98) | 7.32  (7.31-  7.33) | 7.28  (5.83-9.07) | 7.37  (7.36-7.38) | 11.86  (10.06-13.93) | 11.36  (11.34-11.37) | 13.89  (11.57-16.57) | 12.62  (12.61-12.64) |
| MN | Ramsey | 12.28  (9.61-15.60) | 11.64  (11.61-11.66) | 8.77  (6.43-11.84) | 8.48  (8.46-  8.50) | 13.03  (10.09-16.68) | 12.55  (12.52-12.57) | 10.35  (7.89-13.46) | 9.71  (9.69-  9.73) | 14.88  (11.17-19.56) | 13.83  (13.80-13.85) |
| MN | St. Louis | 12.58  (8.83-17.60) | 13.14  (13.11-13.17) | 9.74  (6.66-14.02) | 9.74  (9.72-  9.77) | 10.69  (6.93-16.15) | 9.71  (9.68-9.73) | 10.01  (6.46-15.18) | 9.40  (9.38-  9.43) | 12.81  (7.76-20.41) | 12.07  (12.04-12.10) |
| MN | Washington | 8.50  (5.28-13.40) | 8.73  (8.69-8.77) | 5.20  (2.86-9.28) | 5.23  (5.20-  5.25) | 5.17  (2.70-9.69) | 5.34  (5.31-5.37) | 6.14  (3.79-9.79) | 6.31  (6.28-  6.34) | 7.68  (4.61-12.53) | 8.26  (8.22-8.30) |
| MO | Jackson | 17.77  (14.28-21.90) | 17.96  (17.93-17.99) | 10.30  (7.57-13.87) | 10.95  (10.93-10.97) | 10.29  (7.59-13.82) | 10.51  (10.49-10.53) | 16.18  (12.75-20.32) | 15.40  (15.38-15.43) | 21.44  (17.08-26.56) | 20.58  (20.55-20.62) |
| MO | St. Louis | 16.26  (12.8-20.40) | 16.23  (16.21-16.25) | 9.26  (6.68-12.69) | 9.81  (9.79-  9.82) | 9.46  (6.86-12.91) | 9.42  (9.40-9.43) | 13.82  (10.59-17.85) | 13.00  (12.98-13.02) | 15.63  (11.97-20.15) | 14.46  (14.43-14.48) |
| MT | Gallatin | 7.30  (5.19-10.20) | 9.56  (9.52-9.59) | 6.54  (4.65-9.13) | 8.34  (8.30-  8.37) | 6.55  (4.53-9.40) | 7.02  (6.99-7.04) | 11.00  (8.26-14.49) | 11.16  (11.12-11.19) | 16.02  (12.39-20.46) | 15.87  (15.82-15.92) |
| MT | Hill | 21.87  (18.11-26.20) | 22.02  (21.97-22.07) | 13.18  (10.15-16.94) | 13.38  (13.34-13.42) | 12.06  (8.99-15.98) | 10.98  (10.94-11.01) | 15.29  (12.00-19.29) | 14.07  (14.03-14.11) | 13.01  (9.52-17.53) | 14.33  (14.28-14.38) |
| MT | Lake | 18.22  (15.37-21.50) | 16.47  (16.44-16.50) | 14.53  (11.94-17.58) | 12.83  (12.80-12.86) | 9.46  (7.20-12.34) | 8.95  (8.92-8.97) | 13.51  (10.63-17.01) | 12.95  (12.92-12.98) | 18.83  (14.79-23.67) | 19.61  (19.57-19.66) |
| MT | Missoula | 11.98  (8.84-16.00) | 14.21  (14.18-14.25) | 11.11  (7.91-15.38) | 12.33 (12.29-12.36) | 7.28  (5.09-10.32) | 7.67  (7.64-7.69) | 14.86  (11.30-19.30) | 13.89  (13.85-13.93) | 21.16  (16.71-26.41) | 20.46  (20.40-20.51) |
| MT | Silver Bow | 19.12  (15.50-23.40) | 19.31  (19.25-19.36) | 15.47  (12.08-19.60) | 15.12  (15.07-15.17) | 10.15  (7.21-14.12) | 9.91  (9.87-9.95) | 13.71  (10.10-18.33) | 13.40  (13.35-13.44) | 20.07  (14.94-26.41) | 19.31  (19.24-19.38) |
| MT | Yellowstone | 17.39  (14.54-20.70) | 18.91  (18.88-18.95) | 11.18  (8.97-13.85) | 12.28  (12.25-12.31) | 10.04  (7.84-12.78) | 9.73  (9.70-9.75) | 12.88  (10.46-15.76) | 12.48  (12.46-12.51) | 18.90  (15.32-23.08) | 18.15  (18.11-18.19) |
| NE | Dakota | 22.69  (18.17-28.00) | 19.78  (19.75-19.82) | 9.59  (6.73-13.50) | 10.04  (10.01-10.06) | 6.99  (4.45-10.81) | 7.30  (7.28-7.33) | 10.35  (7.51-14.10) | 8.10  (8.08-  8.12) | 24.50  (18.59-31.56) | 13.98  (13.94-14.01) |
| NE | Douglas | 14.55  (12.39-17.00) | 14.51  (14.49-14.53) | 8.17  (6.61-10.07) | 8.48 (8.46-  8.50) | 9.09  (7.46-11.03) | 8.82  (8.80-8.84) | 15.62  (13.39-18.15) | 14.67  (14.65-14.69) | 20.86  (17.92-24.14) | 19.02  (19.00-19.05) |
| NE | Lancaster | 11.45  (9.79-13.40) | 11.40  (11.38-11.43) | 7.37  (6.07-8.92) | 7.48  (7.46-  7.50) | 8.72  (7.18-10.56) | 8.60  (8.58-8.62) | 13.27  (11.47-15.30) | 12.73  (12.71-12.76) | 18.17  (15.63-21.01) | 16.74  (16.71-16.78) |
| NE | Lincoln | 16.96  (13.46-21.20) | 17.42  (17.37-17.46) | 8.13  (6.02-10.90) | 8.86  (8.83-  8.89) | 7.69  (5.12-11.4) | 7.53  (7.50-7.56) | 12.76  (9.52-16.90) | 11.54  (11.50-11.58) | 19.19  (14.16-25.48) | 17.67  (17.61-17.73) |
| NE | Sarpy | 9.25  (6.88-12.30) | 9.96  (9.92-10.00) | 6.54  (3.81-11.00) | 7.10  (7.07-  7.13) | 8.00  (5.33-11.86) | 7.94  (7.90-7.97) | 10.51  (7.62-14.32) | 10.44  (10.40-10.49) | 10.42  (7.05-15.16) | 10.22  (10.17-10.27) |
| NE | Scotts Bluff | 20.16  (16.41-24.50) | 18.48  (18.44-18.53) | 15.36  (11.90-19.61) | 14.36  (14.31-14.40) | 9.08  (6.66-12.28) | 8.80  (8.76-8.83) | 20.08  (15.94-24.98) | 16.85  (16.80-16.89) | 22.17  (17.29-27.97) | 16.76  (16.71-16.82) |
| NV | Clark | 17.04  (14.60-19.80) | 17.48  (17.47-17.50) | 9.56  (7.79-11.67) | 10.56  (10.55-10.58) | 10.20  (8.25-12.55) | 9.96  (9.95-9.98) | 17.31  (14.73-20.24) | 16.82  (16.80-16.83) | 28.80  (25.20-32.68) | 27.71  (27.68-27.73) |
| NV | Washoe | 18.04  (15.67-20.70) | 17.65  (17.62-17.68) | 11.02  (9.41-12.88) | 12.18  (12.15-12.21) | 11.38  (9.14-14.08) | 11.10  (11.07-11.12) | 17.06  (14.91-19.46) | 16.60  (16.57-16.62) | 22.80  (19.89-25.99) | 21.94  (21.90-21.97) |
| NH | Hills | 11.07  (9.39-13.00) | 11.33  (11.31-11.35) | 9.43  (7.84-11.30) | 9.56  (9.54-  9.57) | 10.05  (8.24-12.21) | 10.14  (10.13-10.16) | 11.93  (9.99-14.18) | 11.46  (11.44-11.48) | 14.74  (12.12-17.82) | 14.34  (14.31-14.36) |
| NH | Merrimack | 13.19  (10.38-16.60) | 13.42  (13.40-13.45) | 10.72  (8.15-13.99) | 10.80  (10.78-10.82) | 10.98  (8.47-14.13) | 10.54  (10.52-10.56) | 13.91  (10.79-17.74) | 12.69  (12.67-12.72) | 14.80  (11.27-19.18) | 15.09  (15.06-15.12) |
| NH | Rockingham | 10.18  (8.18-12.60) | 10.69  (10.68-10.71) | 8.85  (6.72-11.56) | 9.35  (9.33-  9.36) | 9.52  (7.45-12.09) | 9.96  (9.94-9.97) | 10.44  (8.31-13.04) | 10.79  (10.77-10.80) | 13.02  (10.05-16.70) | 13.59  (13.57-13.61) |
| NH | Strafford | 13.67  (10.72-17.30) | 13.57  (13.51-13.62) | 8.95  (6.62-11.99) | 9.54  (9.50-  9.58) | 11.35  (8.25-15.41) | 11.17  (11.13-11.21) | 14.08  (10.53-18.57) | 13.45  (13.40-13.50) | 17.05  (12.79-22.37) | 16.02  (15.96-16.08) |
| NJ | Atlantic | 15.76  (12.85-19.20) | 15.03  (15.01-15.06) | 11.24  (8.77-14.29) | 11.28  (11.26-11.30) | 13.53  (10.56-17.18) | 12.30  (12.28-12.33) | 16.10  (13.03-19.74) | 12.65  (12.63-12.67) | 21.87  (17.66-26.77) | 16.86  (16.83-16.89) |
| NJ | Bergen | 16.23  (12.51-20.80) | 15.16  (15.14-15.17) | 7.81  (5.52-10.93) | 7.91  (7.89-  7.92) | 6.41  (4.38-9.27) | 6.98  (6.96-6.99) | 16.07  (12.36-20.63) | 13.61  (13.59-13.62) | 20.32  (15.68-25.92) | 17.48  (17.46-17.50) |
| NJ | Burlington | 16.19  (12.70-20.40) | 15.39  (15.37-15.42) | 11.29  (8.21-15.32) | 10.88  (10.86-10.91) | 10.38  (7.45-14.30) | 9.94  (9.92-9.96) | 12.77  (9.53-16.91) | 11.97  (11.96-11.99) | 10.78  (7.55-15.16) | 10.64  (10.62-10.67) |
| NJ | Camden | 21.65  (17.68-26.20) | 19.94  (19.91-19.97) | 12.19  (9.35-15.74) | 11.91  (11.88-11.93) | 11.15  (8.51-14.47) | 10.53  (10.50-10.55) | 13.42  (10.14-17.54) | 12.97  (12.95-12.99) | 21.76  (16.72-27.81) | 19.30  (19.27-19.33) |
| NJ | Cumberland | 18.23  (14.38-22.80) | 15.57  (15.54-15.59) | 11.70  (8.83-15.34) | 10.90  (10.87-10.92) | 9.95  (7.32-13.38) | 9.51  (9.49-9.53) | 14.36  (10.72-18.97) | 12.20  (12.18-12.22) | 20.14  (15.65-25.53) | 13.94  (13.91-13.97) |
| NJ | Essex | 19.29  (16.46-22.50) | 18.68  (18.66-18.70) | 11.42  (8.96-14.43) | 11.06  (11.05-11.08) | 10.97  (8.57-13.94) | 10.55  (10.53-10.56) | 18.98  (16.06-22.29) | 17.29  (17.27-17.30) | 24.87  (21.04-29.13) | 21.87  (21.85-21.90) |
| NJ | Gloucester | 13.09  (9.91-17.10) | 13.37  (13.34-13.40) | 7.53  (5.33-10.54) | 8.05  (8.02-  8.07) | 10.65 (7.62-14.70) | 10.24  (10.21-10.26) | 11.56  (7.95-16.52) | 11.67  (11.65-11.70) | 14.07  (9.46-20.41) | 12.93  (12.89-12.96) |
| NJ | Hudson | 22.13  (18.69-26.00) | 20.38  (20.35-20.40) | 11.98  (9.39-15.15) | 11.29  (11.27-11.31) | 10.66  (8.15-13.82) | 10.13  (10.11-10.14) | 21.53  (17.95-25.60) | 19.47  (19.45-19.49) | 29.36  (24.58-34.64) | 26.87  (26.84-26.90) |
| NJ | Hunterdon | 8.12  (5.62-11.60) | 9.68  (9.65-9.71) | 5.31  (3.09-8.97) | 6.24  (6.21-  6.26) | 5.69  (3.77-8.48) | 6.77  (6.74-6.79) | 7.26  (4.57-11.34) | 9.59  (9.56-  9.61) | 9.71  (6.42-14.42) | 10.07  (10.02-10.11) |
| NJ | Mercer | 13.2  (10.32-16.70) | 13.81  (13.75-13.87) | 5.96  (4.15-8.49) | 7.23  (7.18-  7.27) | 6.78  (4.98-9.19) | 7.53  (7.48-7.57) | 12.09  (8.87-16.26) | 12.45  (12.39-12.50) | 16.77  (12.15-22.68) | 15.36  (15.27-15.44) |
| NJ | Middlesex | 17.90  (14.35-22.10) | 16.41  (16.39-16.43) | 12.23  (9.13-16.2) | 11.51  (11.49-11.53) | 10.12  (7.47-13.58) | 9.91  (9.89-9.92) | 14.44  (11.46-18.03) | 13.31  (13.29-13.33) | 20.95  (16.88-25.70) | 18.26  (18.24-18.28) |
| NJ | Monmouth | 12.85  (9.59-17.00) | 13.66  (13.65-13.68) | 6.93  (5.01-9.51) | 7.81  (7.80-  7.83) | 7.96  (5.17-12.07) | 8.40  (8.39-8.42) | 14.22  (10.77-18.55) | 13.16  (13.14-13.18) | 12.79  (9.45-17.09) | 13.35  (13.33-13.37) |
| NJ | Morris | 10.53  (9.59-17.00) | 11.23  (11.21-11.25) | 6.41  (4.54-8.98) | 6.96  (6.95-  6.98) | 6.09  (4.09-8.96) | 6.84  (6.83-6.86) | 12.80  (9.74-16.64) | 11.84  (11.83-11.86) | 12.21  (9.00-16.36) | 11.24  (11.22-11.26) |
| NJ | Ocean | 16.28  (12.63-20.70) | 16.04  (16.02-16.06) | 10.12  (7.59-13.37) | 10.08  (10.07-10.10) | 8.88  (6.40-12.20) | 8.75  (8.73-8.77) | 13.39  (10.30-17.22) | 11.18  (11.16-11.20) | 17.38  (13.25-22.47) | 14.62  (14.60-14.65) |
| NJ | Passaic | 20.18  (16.14-24.90) | 18.85  (18.82-18.88) | 13.05  (9.46-17.73) | 12.33  (12.31-12.36) | 8.69  (6.25-11.94) | 8.69  (8.67-8.71) | 21.01  (16.86-25.86) | 17.90  (17.88-17.93) | 25.59  (20.53-31.41) | 22.47  (22.43-22.51) |
| NJ | Somerset | 12.86  (9.20-17.70) | 12.90  (12.87-12.92) | 6.58  (4.38-9.78) | 7.35  (7.33-  7.37) | 6.61  (4.11-10.48) | 7.52  (7.50-7.54) | 12.23  (8.76-16.82) | 12.03  (12.01-12.05) | 8.19  (5.16-12.76) | 9.19  (9.17-9.22) |
| NJ | Union | 17.89  (14.18-22.30) | 17.36  (17.33-17.38) | 8.28  (6.06-11.22) | 8.56  (8.54-  8.58) | 11.94  (8.75-16.10) | 11.18  (11.16-11.20) | 18.90  (14.79-23.83) | 16.77  (16.75-16.80) | 24.25  (19.42-29.83) | 21.59  (21.55-21.62) |
| NM | Bernalillo | 19.45  (16.82-23.38) | 20.09  (20.07-20.12) | 11.14  (9.32-13.27) | 12.27  (12.25-12.29) | 11.04  (9.05-13.41) | 11.02  (11.00-11.03) | 15.26  (13.16-17.62) | 14.43  (14.42-14.45) | 23.13  (19.85-26.77) | 22.42  (22.39-22.44) |
| NM | Dona Ana | 24.37  (21.23-27.82) | 24.57  (24.52-24.62) | 12.50  (10.37-14.99) | 13.20  (13.16-13.24) | 11.51  (9.15-14.39) | 11.35  (11.32-11.39) | 21.72  (18.49-25.35) | 20.67  (20.62-20.72) | 28.00  (23.80-32.62) | 27.34  (27.28-27.40) |
| NM | San Juan | 19.00  (16.39-21.93) | 21.66  (21.62-21.70) | 10.18  (8.40-12.28) | 11.10  (11.07-11.13) | 11.08  (9.02-13.55) | 11.17  (11.14-11.20) | 16.41  (13.84-19.35) | 16.65  (16.62-16.69) | 21.12  (17.68-25.03) | 19.75  (19.71-19.79) |
| NM | Santa Fe | 16.91  (13.61-20.81) | 17.70  (17.66-17.74) | 10.02  (7.74-12.88) | 11.45  (11.42-11.48) | 8.79  (6.64-11.57) | 9.28  (9.25-9.31) | 18.17  (14.96-21.89) | 17.52  (17.48-17.57) | 27.99  (23.59-32.86) | 26.62  (26.57-26.68) |
| NY | Bronx | 27.60  (23.15-32.50) | 26.98  (26.96-27.01) | 15.62  (12.17-19.84) | 14.75  (14.73-14.77) | 16.36  (11.81-22.22) | 15.29  (15.27-15.31) | 17.21  (12.99-22.46) | 17.25  (17.23-17.27) | 22.82  (17.34-29.41) | 22.50  (22.47-22.53) |
| NY | Kings | 21.97  (18.65-25.70) | 21.54  (21.53-21.56) | 11.80  (9.10-15.16) | 11.58  (11.57-11.59) | 11.19  (8.59-14.45) | 10.94  (10.93-10.95) | 17.88  (14.58-21.73) | 16.49  (16.48-16.50) | 20.16  (16.80-24.00) | 18.82  (18.81-18.84) |
| NY | New York | 17.56  (14.39-21.30) | 17.73  (17.71-17.75) | 10.16  (7.91-12.95) | 10.75  (10.74-10.76) | 10.12  (7.87-12.92) | 10.40  (10.39-10.41) | 15.58  (12.32-19.52) | 14.72  (14.70-14.73) | 17.61  (13.92-22.02) | 16.07  (16.06-16.09) |
| NY | Queens | 20.49  (17.01-24.50) | 20.19  (20.17-20.20) | 11.35  (8.70-14.68) | 11.88  (11.87-11.89) | 8.96  (7.00-11.40) | 9.16  (9.15-9.17) | 19.73  (16.37-23.59) | 17.96  (17.94-17.97) | 22.00  (17.99-26.61) | 19.52  (19.50-19.54) |
| NY | Suffolk | 15.82  (12.18-20.30) | 15.53  (15.51-15.54) | 10.71  (7.78-14.57) | 10.64  (10.63-10.65) | 11.06  (8.13-14.88) | 10.57  (10.56-10.59) | 15.58  (11.91-20.12) | 13.64  (13.63-13.66) | 18.48  (13.85)-24.22) | 16.12  (16.10-16.14) |
| NC | Mecklenburg | 14.08  (11.01-17.80) | 14.95  (14.93-14.98) | 9.94  (7.18-13.60) | 10.34  (10.32-10.36) | 10.83  (7.89-14.68) | 11.29  (11.27-11.32) | 21.29  (17.31-25.89) | 20.41  (20.38-20.44) | 25.79  (21.29-30.86) | 23.31  (23.28-23.34) |
| NC | Wake | 13.24  (10.19-17.00) | 14.35  (14.33-14.37) | 7.97  (5.51-11.40) | 8.99  (8.98-  9.01) | 9.76  (7.24-13.02) | 10.14  (10.13-10.16) | 12.07  (9.39-15.37) | 12.72  (12.70-12.73) | 16.56  (13.22-20.55) | 15.65  (15.63-15.67) |
| ND | Burleigh | 14.09  (11.26-17.50) | 15.42  (15.32-15.52) | 9.27  (7.03-12.13) | 10.11  (10.01-10.21) | 7.57  (5.46-10.41) | 6.98  (6.96-6.99) | 7.66  (5.52-10.54) | 6.97  (6.89-  7.05) | 10.39  (7.56-14.13) | 10.10  (9.98-10.22) |
| ND | Cass | 12.63  (10.30-15.40) | 12.72  (12.68-12.76) | 8.52  (6.72-10.74) | 8.41  (8.37-  8.45) | 10.31  (8.01-13.17) | 9.77  (9.72-9.81) | 8.11  (6.19-10.57) | 7.66  (7.63-  7.70) | 12.31  (9.49-15.82) | 12.02  (11.97-12.07) |
| ND | Stark | 13.61  (10.78-17.10) | 15.85  (15.82-15.88) | 9.71  (7.05-13.22) | 11.05  (11.02-11.08) | 6.51  (4.09-10.22) | 6.77  (6.75-6.79) | 5.87  (3.73-9.13) | 6.16  (6.14-  6.18) | 9.03  (6.15-13.08) | 8.94  (8.91-8.97) |
| ND | Ward | 12.44  (9.62-16.00) | 15.81  (15.78-15.84) | 9.02  (6.48-12.41) | 10.61  (10.58-10.64 | 5.46  (3.38-8.69) | 5.91  (5.89-5.93) | 6.78  (4.53-10.01) | 6.41  (6.39-  6.44) | 9.54  (6.40-13.98) | 9.78  (9.75-9.81) |
| ND | Williams | 12.95  (10.16-16.40) | 15.52  (15.49-15.55) | 7.51  (5.10-10.92) | 8.78  (8.76-  8.81) | 6.45  (4.39-9.38) | 6.12  (6.10-6.14) | 8.87  (6.12-12.69) | 7.65  (7.63-  7.68) | 15.81  (11.92-20.69 | 14.55  (14.51-14.59) |
| OH | Cuyahoga | 21.64  (17.82-26.00) | 21.62  (21.60-21.63) | 10.00  (7.59-13.06) | 10.68  (10.67-10.69) | 10.09  (7.64-13.22) | 10.58  (10.57-10.59) | 11.52  (8.78-14.98) | 11.78  (11.77-11.80) | 15.47  (11.66-20.25) | 14.72  (14.70-14.74) |
| OH | Franklin | 17.02  (14.10-20.40) | 17.30  (17.29-17.32) | 9.67  (7.48-12.42) | 10.43  (10.42-10.45) | 13.82  (11.26-16.85) | 13.25  (13.23-13.27) | 16.29  (13.42-19.63) | 15.99  (15.98-16.01) | 15.76  (12.67-19.44) | 14.77  (14.75-14.79) |
| OH | Hamilton | 16.57  (13.26-20.50) | 17.05  (17.03-17.07) | 10.69  (8.11-13.97) | 11.17  (11.15-11.19) | 9.88  (7.23-13.35) | 9.85  (9.83-9.86) | 18.23  (14.53-22.62) | 17.23  (17.21-17.25) | 18.89  (14.36-24.44) | 17.31  (17.29-17.34) |
| OH | Lucas | 17.45  (13.83-21.80) | 17.41  (17.38-17.43) | 11.03  (8.37-14.40) | 11.36  (11.35-11.38) | 12.43  (9.03-16.86) | 11.83  (11.82-11.85) | 18.00  (13.59-23.45) | 16.69  (16.67-16.72) | 23.73  (17.16-31.85) | 20.93  (20.90-20.96) |
| OH | Montgomery | 14.56  (11.45-18.40) | 15.22  (15.19-15.24) | 10.75  (8.01-14.27) | 11.09  (11.07-11.12) | 11.48  (8.30-15.65) | 11.43  (11.41-11.45) | 16.20  (11.59-22.18) | 15.63  (15.60-15.65) | 18.68  (12.93-26.21) | 17.44  (17.40-17.47) |
| OH | Summit | 21.05  (16.34-26.70) | 20.92  (20.89-20.95) | 11.88  (8.66-16.10) | 12.25  (12.22-12.27) | 17.74  (12.68-24.26) | 16.89  (16.86-16.91) | 12.02  (8.68-16.40) | 12.36  (12.33-12.38) | 14.19  (9.96-19.80) | 14.29  (14.26-14.32) |
| OK | Oklahoma | 17.88  (15.69-20.29) | 18.22  (18.20-18.25) | 10.92  (9.21-12.90) | 11.72  (11.70-11.74) | 11.88  (9.90-14.20) | 11.90  (11.88-11.92) | 18.53  (15.98-21.39) | 17.19  (17.16-17.21) | 27.61  (24.12-31.40) | 24.86  (24.83-24.90) |
| OK | Tulsa | 18.88  (16.36-21.70) | 19.34  (19.31-19.36) | 12.27  (10.30-14.56) | 12.61  (12.59-12.63) | 14.10  (11.77-16.80) | 14.31  (14.28-14.33) | 16.29  (13.77-19.16) | 16.09  (16.06-16.11) | 22.46  (19.18-26.12) | 21.08  (21.05-21.11) |
| OR | Clackamas | 18.23  (13.79-23.70) | 18.41  (18.36-18.47) | 12.61  (9.52-16.51) | 12.91  (12.87-12.96) | 9.51  (6.84-13.07) | 10.23  (10.19-10.27) | 13.73  (10.21-18.21) | 13.86  (13.81-13.90) | 21.54  (15.91-28.48) | 21.37  (21.31-21.44) |
| OR | Lane | 13.91  (10.54-18.10) | 15.23  (15.20-15.26) | 11.09  (8.59-14.21) | 11.59  (11.57-11.62) | 13.39  (9.95-17.77) | 13.20  (13.17-13.22) | 22.01  (17.22-27.70) | 20.48  (20.45-20.52) | 21.88  (16.30-28.71) | 20.57  (20.53-20.61) |
| OR | Multnomah | 16.56  (13.59-20.00) | 16.64  (16.62-16.66) | 11.45  (9.08-14.33) | 11.72  (11.70-11.74) | 11.99  (9.49-15.05) | 12.08  (12.06-12.10) | 16.32  (13.49-19.61) | 15.52  (15.50-15.54) | 22.50  (18.62-26.92) | 20.88  (20.86-20.91) |
| OR | Washington | 12.65  (9.24-17.10) | 13.40  (13.38-13.43) | 8.21  (5.58-11.91) | 8.76  (8.75-  8.78) | 10.14  (7.43-13.70) | 10.83  (10.81-10.85) | 16.57  (12.15-22.19) | 16.13  (16.10-16.15) | 20.46  (15.01-27.25) | 19.25  (19.22-19.28) |
| PA | Allegheny | 14.98  (12.78-17.50) | 14.98  (14.96-14.99) | 12.00  (9.91-14.46) | 12.10  (12.09-12.12) | 11.29  (9.17-13.83) | 10.98  (10.97-11.00) | 10.67  (8.46-13.38) | 10.27  (10.25-10.28) | 11.44  (8.92-14.55) | 11.01  (10.99-11.02) |
| PA | Philadelphia | 20.17  (17.48-23.20) | 20.55  (20.53-20.57) | 11.13  (9.28-13.3) | 11.43  (11.41-11.44) | 14.44  (11.85-17.48) | 13.82  (13.81-13.84) | 16.75  (14.20-19.66) | 15.35  (15.33-15.36) | 19.73  (16.49-23.42) | 19.18  (19.16-19.20) |
| RI | Kent | 12.28  (9.96-15.10) | 12.62  (12.58-12.66) | 11.28  (8.90-14.19) | 10.90  (10.87-10.93) | 10.47  (8.25-13.21) | 10.47  (10.44-10.49) | 12.72  (10.16-15.81) | 12.30  (12.27-12.33) | 13.99  (10.81-17.93) | 14.18  (14.15-14.22) |
| RI | Providence | 18.64  (9.96-15.10) | 17.78  (17.76-17.80) | 10.87  (9.66-12.20) | 10.67  (10.65-10.69) | 10.99  (9.61-12.53) | 10.64  (10.63-10.66) | 16.05  (14.38-17.89) | 14.60  (14.58-14.61) | 21.33  (19.15-23.67) | 18.99  (18.96-19.01) |
| RI | Washington | 12.54  (9.75-16.00) | 13.14  (13.10-13.18) | 9.64  (7.55-12.25) | 9.95  (9.91-  9.98) | 9.61  (7.11-12.86) | 10.28  (10.25-10.31) | 10.14  (7.63-13.34) | 11.07  (11.04-11.11) | 14.68  (10.71-19.78) | 13.87 (13.83-13.91) |
| SC | Beaufort | 10.51  (7.90-13.90) | 11.62  (11.59-11.66) | 10.19  (6.98-14.66) | 10.89  (10.86-10.92) | 7.75  (5.11-11.58) | 7.99  (7.96-8.02) | 16.94  (12.35-22.80) | 16.81  (16.77-16.85) | 26.83  (19.94-35.05) | 25.85  (25.78-25.91) |
| SC | Charleston | 15.73  (12.55-19.50) | 16.47  (16.45-16.49) | 9.51  (7.17-12.51) | 10.68  (10.66-10.70) | 9.23  (6.86-12.32) | 9.48  (9.46-9.49) | 19.82  (16.19-24.03) | 19.38  (19.36-19.41) | 24.85  (20.26-30.07) | 23.40  (23.38-23.43) |
| SC | Greenville | 16.86  (13.3-21.10) | 17.37  (17.35-17.40) | 10.93  (8.42-14.07) | 11.64  (11.62-11.66) | 12.95  (9.66-17.14) | 12.73  (12.70-12.75) | 15.37  (12.18-19.22) | 15.16  (15.13-15.18) | 20.41  (15.98-25.70) | 19.89  (19.86-19.92) |
| SC | Horry | 21.66  (18.25-25.50) | 22.39  (22.35-22.43) | 13.85  (11.11-17.13) | 14.32  (14.29-14.36) | 11.04  (8.38-14.42) | 11.01  (10.98-11.04) | 21.04  (17.36-25.25) | 19.68  (19.64-19.72) | 30.65  (25.63-36.18) | 26.99  (26.94-27.04) |
| SC | Richland | 11.63  (9.24-14.60) | 12.58  (12.55-12.60) | 7.92  (5.83-10.69) | 9.13  (9.11-  9.15) | 8.67  (6.38-11.70) | 9.21  (9.19-9.23) | 14.87  (11.89-18.45) | 14.77  (14.74-14.79) | 22.91  (18.34-28.21) | 22.14  (22.10-22.17) |
| SC | Spartanburg | 20.37  (15.86-25.80) | 21.10  (21.06-21.14) | 13.22  (9.64-17.86) | 13.21  (13.18-13.24) | 8.25  (5.68-11.84) | 8.36  (8.34-8.39) | 19.95  (15.28-25.62) | 18.81  (18.77-18.85) | 23.93  (18.30-30.65) | 22.86  (22.81-22.90) |
| SD | Minnehaha | 9.82  (7.25-13.20) | 10.17  (10.14-10.21) | 5.35  (3.63-7.83) | 6.50  (6.48-  6.53) | 6.45  (4.21-9.75) | 6.43  (6.41-6.46) | 10.74  (7.82-14.58) | 9.69  (9.65-  9.73) | 17.35  (13.17-22.52) | 16.29  (16.24-16.35) |
| SD | Pennington | 14.87  (11.78-18.60) | 14.60  (14.53-14.66) | 8.33  (6.13-11.22) | 8.23  (8.19-  8.28) | 9.21  (6.54-12.82) | 8.65  (8.59-8.70) | 14.91  (11.55-19.04) | 12.97  (12.90-13.04) | 12.06  (8.76-16.37) | 12.41  (12.34-12.48) |
| TX | Bexar | 23.44  (19.59-27.80) | 23.96  (23.94-23.97) | 10.35  (7.87-13.50) | 11.04  (11.03-11.06) | 7.71  (5.47-10.75) | 8.03  (8.02-8.04) | 20.78  (16.94-25.22) | 20.08  (20.07-20.10) | 34.46  (29.30-40.02) | 32.52  (32.50-32.54) |
| TX | Dallas | 18.27  (14.60-22.60) | 18.76  (18.74-18.77) | 10.32  (7.11-14.74) | 10.89  (10.88-10.90) | 9.20  (6.25-13.34) | 8.95  (8.94-8.96) | 23.26  (18.52-28.79) | 21.40  (21.38-21.41) | 34.82  (28.87-41.29) | 31.62  (31.60-31.63) |
| TX | El Paso | 20.11  (16.70-24.00) | 21.94  (21.91-21.96) | 9.84  (7.50-12.80) | 10.47  (10.45-10.48) | 8.91  (6.63-11.87) | 8.82  (8.80-  8.83 | 22.36  (18.56-26.68) | 22.17  (22.14-22.19) | 39.35  (34.01-44.96) | 40.16  (40.13-40.19) |
| TX | Fort Bend | 13.24  (9.39-18.40) | 15.14  (15.11-15.17) | 5.72  (3.51-9.19) | 6.45  (6.43-  6.47) | 5.39  (2.96-9.60) | 6.23  (6.22-6.25) | 14.46  (10.47-19.63) | 14.52  (14.49-14.55) | 18.89  (13.34-26.05) | 18.40  (18.37-18.44) |
| TX | Harris | 17.88  (14.19-22.30) | 17.88  (17.87-17.89) | 7.95  (5.65-11.08) | 8.54  (8.53-  8.55) | 8.85  (6.45-12.02) | 9.05  (9.05-9.06) | 20.68  (16.99-24.92) | 19.71  (19.70-19.72) | 37.35  (32.37-42.60) | 34.40  (34.38-34.41) |
| TX | Hidalgo | 29.55  (24.74-34.90) | 31.06  (31.03-31.09) | 12.99  (10.33-16.20) | 13.63  (13.60-13.65) | 9.21  (6.79-12.36) | 9.24  (9.22-9.26) | 34.33  (29.2-39.85) | 33.01  (32.98-33.04) | 57.52  (50.85-63.92) | 56.11  (56.08-56.15) |
| TX | Lubbock | 19.60  (14.89-25.40) | 20.28  (20.24-20.33) | 9.13  (5.99-13.67) | 10.35  (10.32-10.38) | 9.00  (5.63-14.08) | 8.81  (8.78-8.84) | 20.74  (15.72-26.85) | 18.95  (18.91-18.99) | 26.19  (19.96-33.54) | 23.47  (23.42-23.52) |
| TX | Tarrant | 13.63  (10.35-17.80) | 13.65  (13.63-13.66) | 7.70  (5.40-10.87) | 8.24  (8.23-  8.25) | 9.96  (7.00-13.99) | 9.67  (9.66-9.68) | 15.6  (12.07-19.92) | 14.57  (14.56-14.58) | 28.59  (22.87-35.09) | 26.34  (26.32-26.35) |
| TX | Travis | 15.75  (11.92-20.50) | 16.20  (16.19-16.22) | 5.78  (3.98-8.34) | 6.28  (6.27-  6.29) | 10.78  (7.78-14.75) | 10.35  (10.33-10.36) | 19.17 (15.14-23.96) | 18.36  (18.34-18.38) | 25.49  (20.55-31.14) | 24.12  (24.10-24.14) |
| UT | Cache | 12.93  (9.51-17.40) | 13.57  (13.48-13.65) | 7.25  (4.70-11.03) | 8.41  (8.34-  8.48) | 9.89  (6.68-14.40) | 10.08  (10.01-10.15) | 16.36  (12.60-20.98) | 14.19  (14.13-14.26) | 25.90  (20.39-32.29) | 21.35  (21.24-21.46) |
| UT | Davis | 8.88  (7.13-11.00) | 9.29  (9.27-9.31) | 6.96  (5.48-8.80) | 7.50  (7.48-  7.52) | 8.38  (6.66-10.49) | 8.67  (8.65-8.69) | 13.02  (10.65-15.82) | 13.64  (13.62-13.67) | 10.92  (8.48-13.96) | 10.18  (10.15-10.20) |
| UT | Salt Lake | 13.10  (11.85-14.50) | 12.93  (12.92-12.94) | 9.03  (8.08-10.07) | 9.30  (9.29-  9.31) | 10.16  (9.05-11.39) | 10.06  (10.05-10.07) | 15.33  (13.97-16.79) | 14.74  (14.73-14.75) | 19.39  (17.67-21.23) | 17.31  (17.30-17.33) |
| UT | Utah | 10.51  (8.94-12.30) | 10.69  (10.67-10.70) | 6.99  (5.78-8.45) | 7.73  (7.71-  7.74) | 8.57  (7.08-10.34) | 8.69  (8.68-8.70) | 15.72  (13.74-17.93) | 15.11  (15.09-15.13) | 20.65  (18.04-23.53) | 18.59  (18.57-18.61) |
| UT | Washington | 11.54  (8.63-15.30) | 12.86  (12.75-12.98) | 11.39  (8.52-15.07) | 11.78  (11.66-11.90) | 12.55  (9.38-16.59) | 11.68  (11.57-11.80) | 16.32  (12.70-20.72) | 13.34  (13.25-13.43) | 29.11  (23.37-35.60) | 26.69  (26.48-26.89) |
| UT | Weber | 15.93  (13.37-18.90) | 15.33  (15.29-15.36) | 9.44  (7.58-11.7) | 9.66  (9.63-  9.68) | 11.36  (9.03-14.20) | 11.06  (11.03-11.09) | 17.27  (14.34-20.66) | 15.54  (15.51-15.56) | 18.79  (15.32-22.85) | 17.26  (17.22-17.30) |
| VT | Chittenden | 9.98  (7.96-12.50) | 10.78  (10.75-10.82) | 7.54  (5.94-9.51) | 8.15  (8.12-  8.18) | 8.22  (6.38-10.54) | 8.90  (8.87-8.93) | 7.47  (5.67-9.78) | 7.91  (7.88-  7.93) | 8.78  (6.45-11.83) | 8.81  (8.77-8.84) |
| VT | Rutland | 14.30  (11.36-17.80 | 13.83  (13.79-13.87) | 11.65  (9.01-14.95) | 11.23  (11.19-11.27) | 10.72  (8.21-13.87) | 9.87  (9.83-9.90) | 8.94  (6.72-11.80) | 8.82  (8.79-  8.86) | 15.79  (11.52-21.26) | 15.01  (14.96-15.07) |
| VT | Washington | 12.96  (10.02-16.60) | 13.08  (13.04-13.12) | 9.98  (7.24-13.6) | 10.27  (10.24-10.31) | 10.20  (7.18-14.30) | 9.65  (9.62-9.69) | 9.52 (6.84-13.10) | 9.13  (9.09-  9.16) | 9.80  (6.63-14.24) | 9.73  (9.69-9.77) |
| VT | Windsor | 10.84  (8.30-14.00) | 11.95  (11.91-11.99) | 8.00  (5.89-10.78) | 8.97  (8.94-  9.01) | 9.77  (7.03-13.43) | 9.52  (9.48-9.55) | 9.77 (7.12-13.26) | 9.39  (9.35-  9.42) | 11.47  (7.96-16.26) | 11.01  (10.95-11.06) |
| WA | Clark | 14.96  (11.69-19.00) | 15.13  (15.10-15.15) | 13.44  (10.35-17.27) | 13.45  (13.42-13.48) | 13.11  (9.91-17.14) | 12.68  (12.65-12.71) | 14.33 (10.78-18.80) | 14.06  (14.04-14.09) | 15.68  (11.59-20.86) | 14.62  (14.59-14.65) |
| WA | King | 11.18  (9.74-12.80) | 11.16  (11.15-11.17) | 8.22  (7.00-9.64) | 8.21  (8.20-  8.21) | 10.49  (9.00-12.20) | 10.35  (10.35-10.36) | 13.80 (12.17-15.61) | 13.37  (13.36-13.38) | 16.36  (14.27-18.68) | 15.18  (15.17-15.19) |
| WA | Pierce | 18.51  (15.72-21.70) | 18.37  (18.35-18.39) | 12.27  (9.96-15.03) | 12.30  (12.28-12.32) | 12.40  (9.93-15.37) | 12.14  (12.13-12.16) | 18.75 (15.82-22.08) | 17.88  (17.86-17.89) | 23.34  (19.63-27.51) | 21.52  (21.50-21.55) |
| WA | Snohomish | 18.19  (15.02-21.90) | 18.18  (18.15-18.20) | 13.55  (10.89-16.74) | 13.45  (13.43-13.47) | 11.23  (8.74-14.32) | 11.17  (11.15-11.19) | 16.23  (13.08-19.96) | 16.19  (16.17-16.21) | 16.96  (13.34-21.33) | 15.84  (15.82-15.87) |
| WA | Spokane | 15.40  (12.17-19.30) | 15.45  (15.42-15.47) | 9.04  (6.64-12.20) | 9.20  (9.18-  9.22) | 10.42  (7.68-14.00) | 10.63  (10.61-10.65) | 16.90 (13.29-21.25) | 16.28  (16.26-16.31) | 21.49  (16.92-26.89) | 20.21  (20.18-20.25) |
| WV | Kanawha | 20.77  (17.59-24.40) | 21.86  (21.74-21.98) | 14.16  (11.53-17.27) | 14.97  (14.87-15.07) | 14.21  (11.41-17.57) | 14.34  (14.24-14.43) | 21.54  (17.94-25.65) | 20.69  (20.57-20.82) | 24.30  (19.70-29.58) | 24.47  (24.31-24.64) |
| WI | Milwaukee | 22.21  (17.86-27.30) | 21.93  (21.90-21.97) | 16.52  (12.64-21.30) | 16.23  (16.20-16.26) | 15.03  (10.92-20.32) | 14.46  (14.43-14.48) | 18.75  (14.72-23.56) | 17.58  (17.55-17.61) | 18.99  (14.58-24.36) | 17.78  (17.74-17.81) |
| WY | Laramie | 17.37  (14.45-20.70) | 16.15  (16.10-16.21) | 13.04  (10.34-16.31) | 12.40  (12.35-12.45) | 10.58  (7.79-14.21) | 10.49  (10.44-10.54) | 16.5  (13.19-20.46) | 15.09  (15.03-15.14) | 22.83  (18.36-28.02) | 22.63  (22.55-22.70) |
| WY | Natrona | 17.73  (14.58-21.40) | 18.70  (18.64-18.76) | 11.75  (9.20-14.88) | 12.43  (12.39-12.48) | 12.58  (9.54-16.39) | 12.19  (12.14-12.24) | 16.42  (12.82-20.79) | 15.48  (15.43-15.53) | 27.83  (22.86-33.41) | 25.63  (25.56-25.70) |
| ^a^ Confidence intervals for the BRFSS are produced using the Taylor method of estimation. The BRFSS SAE uses the SAS GLIMMIX procedure which does not take the complex survey design into effect, thereby limiting the magnitude of the confidence intervals. Comparisons of confidence intervals from two different methods should be approached with caution. | | | | | | | | | | | |
